# Human Colorectal Cancer from the Perspective of Mouse Models

**DOI:** 10.3390/genes10100788

**Published:** 2019-10-11

**Authors:** Monika Stastna, Lucie Janeckova, Dusan Hrckulak, Vitezslav Kriz, Vladimir Korinek

**Affiliations:** Institute of Molecular Genetics of the Czech Academy of Sciences, Videnska 1083, 142 20 Prague, Czech Republic; monika.stastna@img.cas.cz (M.S.); lucie.janeckova@img.cas.cz (L.J.); dusan.hrckulak@img.cas.cz (D.H.); vitezslav.kriz@img.cas.cz (V.K.)

**Keywords:** carcinoma, consensus molecular subtypes, intestine, oncogenes, signaling cascades, tumor suppressors, tumorigenesis

## Abstract

Colorectal cancer (CRC) is a heterogeneous disease that includes both hereditary and sporadic types of tumors. Tumor initiation and growth is driven by mutational or epigenetic changes that alter the function or expression of multiple genes. The genes predominantly encode components of various intracellular signaling cascades. In this review, we present mouse intestinal cancer models that include alterations in the Wnt, Hippo, p53, epidermal growth factor (EGF), and transforming growth factor β (TGFβ) pathways; models of impaired DNA mismatch repair and chemically induced tumorigenesis are included. Based on their molecular biology characteristics and mutational and epigenetic status, human colorectal carcinomas were divided into four so-called consensus molecular subtype (CMS) groups. It was shown subsequently that the CMS classification system could be applied to various cell lines derived from intestinal tumors and tumor-derived organoids. Although the CMS system facilitates characterization of human CRC, individual mouse models were not assigned to some of the CMS groups. Thus, we also indicate the possible assignment of described animal models to the CMS group. This might be helpful for selection of a suitable mouse strain to study a particular type of CRC.

## 1. Introduction

Cancer of the colon and rectum (colorectal cancer (CRC)) is one of the most commonly diagnosed cancer types in Western countries. In the United States (US), the lifetime risk of CRC is 5%, and the death rate of diagnosed patients exceeds 30% [1]. Approximately 85% of colorectal tumors arise sporadically, and 15% are underlined by hereditary predispositions (reviewed in Reference [2]). The early stages of colorectal tumors are predominantly associated with mutations in the tumor suppressor adenomatous polyposis coli (*APC*) [3], resulting in aberrant activation of the Wnt signaling pathway. A subsequent mutation usually affects the Kirsten rat sarcoma viral oncogene homolog (*KRAS*) gene, which further enhances Wnt signaling and thereby facilitates the adenoma growth [4,5]. In addition, mutations inactivating tumor protein 53 (encoded by the *TP53* gene) and some of the SMAD (an acronym of *Caenorhabditis elegans sma* and *Drosophila melanogaster* mothers against decapentaplegic genes) family member genes accumulate in the cancer cell; these mutations further promote progression of premalignant intestinal polyps toward carcinomas [6,7,8].

Colitis-associated colorectal cancer (CAC) arises as a result of chronic inflammation in the intestine and accounts for 1–2% of all CRCs (reviewed in Reference [9]). CAC tumors are situated within the colon in the areas of active inflammation and develop similarly to CRC via accumulation of numerous mutations in intestinal epithelial cells (reviewed in Reference [10]). However, while sporadic CRC is underlined by *APC* disruption, the earliest mutation event in CAC mainly affects the *TP53* gene [11]. Nevertheless, as in case of sporadic CRC, early activation of Wnt signaling is critical for the colitis-to-cancer transition [12]. *TP53* mutations were found in up to 89% of CAC patients [13], while other mutations present in sporadic CRC were less frequent, e.g., the *APC* gene alterations were found in less than 30% of CAC specimens [14]. In addition, *KRAS* mutations were detected in approximately 30–40% of both sporadic CRC and CAC [13,15,16]. CAC differs from sporadic CRC not only in the order of acquired mutations, but also by the type of mutations in individual genes. For example, in sporadic CRC, mutations in the *TP53* gene mainly impair the protein ability to bind DNA; however, in CAC such mutations are less frequent. In contrast, several “gain of function” (GOF) alterations of the *TP53* gene that increase tumor invasiveness, attenuate apoptosis, and increase genomic instability were predominantly found in CAC [13,17].

The classification of colorectal tumors underwent significant changes over the last few years. The original approach of CRC classification was based on gene expression analysis, which, however, often showed considerable differences depending on the dataset used and experimental approach employed by individual research groups. To unify the classification of intestinal tumors, Guinney and colleagues performed a large-scale data analysis by linking six previously published CRC subtyping algorithms [18,19,20,21,22,23]. The analysis resulted in the system of four consensus molecular subtypes (CMSs). Individual CMSs were defined not only by gene expression, but also by other characteristics such as mutation counts, somatic copy number alterations (SCNAs), i.e., gain or loss in copies of genomic DNA, microsatellite instability (MSI), cytosine-phosphate diester-guanine nucleotide (CpG) island methylator phenotype (CIMP), and differences in the immune response and activation status of various signaling pathways. The authors created a “gold standard” of CRC classification, where each CMS group is defined by certain biological properties, gene expression profiles, and clinical course [24]. According to this classification, most intestinal tumors (78% of 4151 tumors analyzed) may be assigned to one of the four CMS groups: CMS1 (also named “MSI immune”; 14% cases), CMS2 (“canonical”; 37%), CMS3 (“metabolic”; 13%), and CMS4 (“mesenchymal”; 23%) (Table 1). Tumors from the CMS1 group differed markedly from all other groups by high mutation counts and low SCNA counts, pronounced MSI, and wide-spread DNA hypermethylation. They overexpressed proteins involved in DNA damage repair and frequently carried mutations in the B-Raf proto-oncogene (*BRAF*); however, mutations in *APC*, *TP53*, and *KRAS* often occurred as well. The tumors also exhibited strong immune cell infiltration and activation, they predominantly occurred in the right colon, and patients had a low survival rate after relapse. In contrast, tumors from other groups had elevated SCNA counts, possibly related to high chromosomal instability (CIN). CMS2 group tumors displayed more frequent gains in the copy number of oncogenes and losses in tumor suppressor genes in comparison to other groups displaying CIN, i.e., CMS3 and CMS4 groups. The CMS2 tumors also exhibited elevated epithelial differentiation and hyperactivation of the Wnt pathway and increased Myc-dependent transcription. On the other hand, gene signatures indicating epithelial–mesenchymal transition (EMT) and matrix remodeling were underrepresented. Moreover, CMS2 group patients had tumors distributed within the left colon and rectum and better survival rates than those in other groups. Although tumors from the CMS3 group displayed high CIN in comparison to the CMS2 and CMS4 groups, they showed less SCNA counts and higher CIMP, and they were “hypermutated”. CMS3 group tumors displayed the highest incidence of *KRAS* mutations, which are possibly linked to metabolic deregulation found in this type of CRC. Finally, CMS4 group tumors had typically high SCNA counts, hyperactivated transforming growth factor β (TGFβ) signaling, and increased expression of genes involved in EMT, angiogenesis, and matrix remodeling. Interestingly, CMS4 group tumors as the only group showed a gene expression profile indicating infiltration by both mesenchymal and immune cells. Patients from the CMS4 tumor group had the worst overall and relapse-free survival rates of all CMS groups [24]. Whereas the CMS classification is mainly based on analysis of sporadic CRC, the question arises with regard to how to assign CAC malignancies to the system. Since the initial mutation in CAC affects the *TP53* gene, CAC tumors could be included in the CMS2 or CMS4. However, CMS2 group tumors displayed decreased immune infiltration, which does not correspond to elevated pro-inflammatory nuclear factor kappa-light-chain-enhancer of activated B cells (NF-κB) signaling in CAC. On the other hand, tumors from the CMS1 group showed high immune infiltration and, in addition, CMS4 group neoplasms exhibited robust complement activation that was reported to contribute to CAC in the mouse model [25]. In conclusion, CAC characteristics do not completely fall into any particular CMS group. Moreover, although CAC tumors share multiple mutations with CRCs, the mutations are accumulated in a different order and the tumors develop in a specific microenvironment caused by chronic inflammation. Thus, CAC lies beyond the categorization developed for CRC.

The conclusions of the Guinney et al. study were used by Linnekamp and co-workers, who tested different CRC cell lines and, based on their properties, categorized them into the individual CMS groups [26]. Using different gene expression datasets from publicly available databases, 43 CRC cell lines were classified into individual CMS groups. Although the assignment into a particular CMS group often varied depending on the dataset used, 66% of the CRC cell lines showed consistent assignment to a specific CMS group across the datasets tested. The study also included mutational changes in CRC cell lines and alterations in the status of five major pathways that are frequently deregulated in CRC. The Wnt, p53, and receptor tyrosine kinase (RTK)/Ras pathways displayed similar alterations in CRC cells as in patient samples, whereas phosphatidylinositol-3-kinase (PI3K) and TGFβ pathways were mutated in CRC-derived cell lines with significantly lower frequencies than in tumor specimens. Furthermore, 18 cell lines were grown as xenografts and, even after multiple transfer, the cells maintained the original gene expression profiles. Moreover, cells isolated from 33 CRC patients were cultured in vitro as organoids. Interestingly, according to gene expression, organoids might also be classified into the four CMS groups. Importantly, with one exception, organoids retained the same expression patterns observed in the original tumor specimens [26]. Studies of CRC specimens, CRC-derived cell lines, and organoids brought considerable simplification, clarification, and unification of CRC characterization. However, elucidation of the molecular mechanisms involved in tumor initiation and progression requires analysis in living organisms. Although several recent articles provided an overview of mouse models suitable for studying CRC [27,28], individual mouse models and strains remain to be assigned to the particular CMS group. Since CRC is a highly heterogeneous disease and the individual tumor subtypes display various characteristics, it is important to select the right preclinical model to best mimic the human disease and thereby reduce misleading conclusions. Therefore, in the following chapters, mouse models that are broadly used to study mutations frequently observed in human CRC are discussed, and the possible assignment of a specific cancer model to some of the CMS group(s) is suggested. We anticipate that many of the mouse models do not easily align with the established CMS classification. Nevertheless, for each CMS group, mouse strains that best fit the group characteristics are summarized in Table 2. Finally, the best studied so-called canonical branches of the particular signaling pathways are discussed throughout the review. These pathways are believed to function in an analogous manner in both human and mouse. Nonetheless, species-specific differences are indicated when appropriate.

## 2. Mouse Models of Chemically Induced Colorectal Tumorigenesis

Since different chemical compounds cause different mutations, utilization of chemical mutagens results in generation of a variety of tumors that fall into all CMS groups. Consequently, chemically induced tumors mimic the wide range of genetic alterations found in sporadic CRC and CAC. Additionally, chemical induction of intestinal tumors can be used to study the tumorigenic properties of chemical substances commonly found in the human diet or environment. One group of such chemical substances are heterocyclic aromatic amines that are present in grilled or roasted meat. For example, 2-amino-1-methyl-6-phenylimidazol[4,5-b] pyridine (PhIP) was used several times to induce tumors in the mouse or rat colons; however, the tumor incidence was relatively low [38,39], although the tumor incidence was increased when PhIP treatment was combined with a high-fat diet [40]. Other tumorigenic substances are alkylnitrosamide compounds such as methylnitrosourea. This topical carcinogen does not require metabolic activation and, thus, may be administered directly into the colon lumen. Tumors induced by methylnitrosourea are formed mainly in the distal colon and rectum [41]. The lesions are well differentiated and frequently invade the submucosa. However, tumor induction by intrarectal administration of the mutagen is not high, and reproducibility of such experiments depends on the skill of the experimenter (reviewed in Reference [42]). The most frequently used chemicals for CRC induction are 1,2-dimethylhydrazine (DMH) [43] or its metabolite azoxymethane (AOM). AOM is a potent carcinogen that causes a wide spectrum of mutations in key genes encoding components of multiple intracellular signal transduction cascades [44,45,46,47]. Upon administration, AOM is metabolized to methylazoxymethanol, and subsequently to formaldehyde and a methyldiazonium ion. The latter is highly reactive and causes alkylation of DNA bases. Repetitive administration of AOM leads to development of epithelial neoplasia initiated by abnormal colonic crypts, so-called aberrant crypt foci (ACF); ACF further progress to adenoma and malignant adenocarcinoma [48]. AOM-treated mice generate tumors predominantly in the distal colon; the tumors reach the advanced carcinoma stage within a few months after the mutagen administration. This can be considered an advantage, since the majority of genetic mouse models—in contrast to humans—produce tumors mainly in the small intestine. Moreover, as described in the following chapters, genetic manipulations of tumor suppressors or oncogenes predominantly induce multiple tumors that severely disturb the absorptive function of the epithelium. The tumor burden leads to preconscious animal death before individual tumors reach advanced stages [49]. Interestingly, it was reported that various laboratory mouse strains displayed different sensitivity to AOM (the sensitivity is manifested by the number of induced lesions) [50].

To create a model of colorectal tumors associated with chronic inflammation, a protocol combining AOM with an inflammatory agent, dextran sulfate sodium (DSS) salt, was introduced. Chronic inflammation leads to the formation of a microenvironment enriched with immune cells that produce pro-inflammatory cytokines and growth factors and, simultaneously, increase the local levels of reactive oxygen species. Subsequently, cell proliferation and the risk of DNA damage are increased. In the case of a long-lasting inflammatory response, cell transformation and tumorigenesis occur with high frequency. The inflammatory response and cell survival are promoted by the NF-κB signaling pathway. As shown in mice with conditional deletion of IκB kinase β (*IKKβ*), impairment of NF-κB signaling in colonic epithelial cells led to decreased tumor incidence without affecting the level of inflammation in AOM/DSS-treated mice [51]. Another advantage of the AOM and DSS combination is further reduction in the time needed for tumor formation. A single dose of AOM followed by five days of DSS treatment resulted in development of multiple colon tumors within 10 weeks [52,53]. This procedure proved to be very reliable and reproducible and was used to induce CAC in mice. Given the different mutation site in genes such as *Ctnnb1* (the *Ctnnb1* gene encodes β-catenin), it is evident that the combination of AOM and an inflammatory agent induces a different spectrum of tumors in comparison to induction by the carcinogen alone (reviewed in Reference [54]; all indicated models of chemically induced colorectal tumorigenesis are summarized in Table S1, Supplementary Materials).

## 3. Mouse Models of Aberrant Wnt Signaling

The canonical (i.e., β-catenin-dependent) Wnt signaling pathway maintains the balance between proliferation and differentiation of intestinal epithelial cells. Consequently, mutations resulting in aberrant activation of Wnt signaling initiate and promote tumorigenesis. Tumor suppressor gene *APC* encodes a key negative regulator of the pathway and it represents the most frequently mutated gene in CRC (reviewed in Reference [55]). Mutations in *APC* occur in all CMS tumor groups, with the highest representation in CMS2 (83%) and the lowest in CMS1 (40%). Concordantly, hyperactivation of the canonical Wnt signaling pathway was observed predominantly in CMS2 group tumors [24]. This chapter presents mouse models carrying (inducible) mutations in the *Apc* and *Ctnnb1* genes, as well as models enabling hyperactivation of the Wnt pathway by Wnt agonists R-spondins (RSPOs; corresponding models are summarized in Table S2, Supplementary Materials).

**Figure 1:**
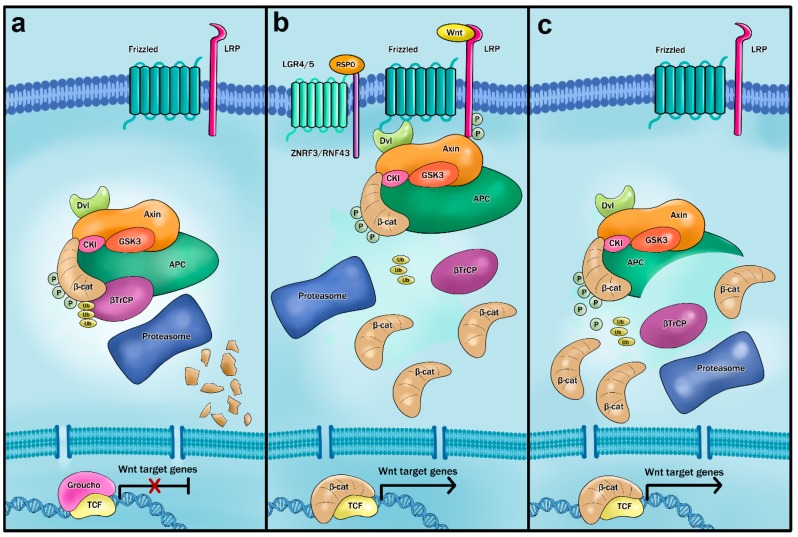
The canonical Wnt signaling pathway. (**a**) In the absence of Wnt ligand, a cytosolic protein complex composed of adenomatous polyposis coli (APC), axis inhibition (Axin), casein kinase 1 α (CK1α), glycogen synthase kinase 3 β (GSK3β), and β-transducin repeat-containing E3 ubiquitin protein ligase (βTrCP) mediates phosphorylation and ubiquitination of β-catenin (β-cat). Phosphorylated β-catenin is subsequently degraded by the proteasome. In such a situation, transcription factors from the T-cell factor/lymphoid enhancer-binding factor (TCF/LEF) family are held in an inactive state by interaction with transcription repressor Groucho that blocks transcription of Wnt signaling target genes. (**b**) Binding of the Wnt ligand to receptor Frizzled and co-receptor low-density lipoprotein receptor-related protein (LRP) leads to LRP phosphorylation that induces Axin recruitment to the cell membrane. As a result, the destruction complex is disassembled and β-catenin translocates to the cell nucleus to activate, in cooperation with TCF/LEF factors, transcription of Wnt target genes. R-spondin (RSPO) ligand binds the leucine-rich repeat-containing G-protein coupled receptor (Lgr) 4/5, which results in internalization and subsequent proteasomal degradation of transmembrane E3 ubiquitin ligases zinc and ring finger 3 (ZNRF3) and ring finger 43 (RNF43). The ligases mediate turnover of the Wnt receptor Frizzled and their inhibition enhances Wnt signaling. (**c**) Truncated APC protein does not retain the ability to scaffold the destruction complex, resulting in β-catenin stabilization and aberrant expression of Wnt target genes, i.e., even without the presence of the Wnt ligand.

Wnt signaling is initiated upon Wnt ligand binding to the cell surface receptor Frizzled and co-receptor low-density lipoprotein receptor-related protein 5/6 (LRP5/6). The binding initiates a cascade of events leading to disintegration of the so-called β-catenin destruction complex, a cytosolic protein complex that regulates β-catenin stability (reviewed in Reference [56]). The APC protein interacts with β-catenin and establishes a protein core for the destruction complex, which further contains glycogen synthase kinase 3β (GSK3β), casein kinase 1 (CK1), and scaffold proteins axis inhibition 1 and 2 (AXIN1 and AXIN2) (reviewed in References [57,58,59]). Mutations in the *APC* or *CTNNB1* genes prevent formation of the destruction complex. This results in β-catenin stabilization and β-catenin entry into the nucleus. Nuclear β-catenin, together with transcription factors from the T-cell factor/lymphoid enhancer-binding factor (TCF/LEF) family, activates transcription of genes important for cell proliferation and cell survival [60,61,62] (Figure 1). Approximately 90% of sporadic colorectal tumors carry a mutation in *APC* and up to 5% in the *CTNNB1* gene (reviewed in References [63,64]). Relatively rare are mutations in Wnt negative regulators *AXIN1/2* and in transcription factor *TCF4* (reviewed in References [65,66]).

**Figure 2:**
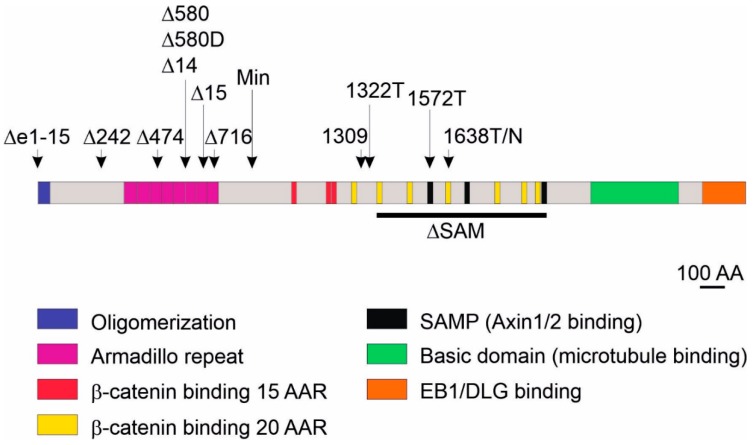
The domain structure and truncated variants of mouse adenomatous polyposis coli (Apc) protein. The scheme indicates positions of germline *Apc* mutations utilized in mouse models. The names of mutations correspond to the terms used in the studies describing a particular cancer model; the region which was deleted in the *Apc^ΔSAM^* allele is underlined; Δ indicates deletion; AA, amino acid; AAR, amino-acid repeats; Axin, Axis inhibition; DLG, discs large; EB1, end-binding protein 1; SAMP, serine–alanine–methionine–proline.

The *APC* locus was discovered by studying a rare hereditary syndrome, familial adenomatous polyposis (FAP) [67,68]. Inherited mutation in the *APC* gene leads to development of hundreds to thousands adenomatous polyps predominantly located in the colon and rectum; the occurrence of polyps in the small intestine is less common. Because of frequent random inactivation of the second *APC* allele and successive accumulation of additional tumor-promoting mutations, the polyps progress to carcinoma by the age of 35 (reviewed in Reference [69]). Since most colorectal tumors harbor a mutation in the *APC* gene, a large proportion of mouse genetic intestinal cancer models target (or involve) the *Apc* gene (Figure 2).

The human APC protein consists of 2843 amino acids, and its interactions with other proteins of the β-catenin destruction complex are mediated by several domains (amino-acid repeats) located in the central part of the protein. There are three 15-amino-acid repeats (15AARs) that bind β-catenin constitutively and seven 20-amino-acid repeats (20AARs) that bind β-catenin inducibly (the interaction with 20AARs depends on the phosphorylation status of β-catenin) [70]. Three serine–alanine–methionine–proline (SAMP) amino-acid repeats are responsible for interactions with AXIN1/2 [71]. The N-terminal part of APC contains another protein interaction domain that includes eight so-called armadillo repeats. Finally, the C-terminus of the protein interacts with proteins involved in microtubule assembly, cell polarity, and chromosome segregation. More than 60% of *APC* mutations are located in a mutation cluster region (MCR) in exon 15, and, in most cases, the mutations result in loss of the C-terminal portion of APC protein [72,73]. The amino-acid sequence and domain composition of the Apc protein is evolutionarily conserved in metazoan species ranging from *Drosophila* to humans [74]. As the sequence identity of the human and mouse Apc proteins exceed 89%, the mouse represents a suitable mode to study the involvement of Apc truncations in intestinal cancer.

### 3.1. Multiple Intestinal Neoplasia (*Min*) Mice

The *Apc^+/Min^* mouse strain is a frequently used genetic mouse model to study CRC. Similarly to FAP patients, these mice (generated by random chemically induced mutagenesis) carry a nonsense germline mutation in one *Apc* allele that results in *Apc* truncation at codon 851 [32,75]. The Min mutation is autosomal dominant with 100% penetrance; while at homozygote state the mutation is embryonically lethal, the heterozygote animals are viable. After random inactivation of the second allele, adult *Apc^+/Min^* mice develop multiple intestinal polyps. The polyps predominantly develop in the small intestine, and to a much lesser extent in the colon. Occasionally, tumors might also appear in the mammary glands and stomach [76,77]. Importantly, the incidence of polyps is dependent on the genetic background and may be influenced by the diet. For example, intestinal polyps developed with 100% penetrance in *Apc^+/Min^* mice on the C57BL/6 background do not progress to carcinoma as the animals die at young age (16 to 18 weeks) due to anemia, inflammation, and other symptoms associated with digestive tract damage. In addition, the mice developed a large number of small intestinal tumors and a relatively low number of tumors in the colon [75]. In contrast, only 7% tumor incidence was observed in *Apc^+/Min^* mice of the FVB/Nj genetic background [78]. Recently, Sodrig and colleagues reported extensive colon carcinogenesis in *Apc^+/Min^* mice of the AKR/J background [79]. Strikingly, Cooper and co-workers documented that the presence of the *Min* allele in the animals (presumably) of the same genetic background but originating from separate colonies might be manifested by a remarkably differing phenotype. The authors of the study purchased *Apc*^+/*Min*^ males of the C57BL/6 background from the Jackson Laboratory and crossed them with the wild-type (wt) C57BL/6 females originating from the Jackson Laboratory or from the domestic facility. The animals of the latter mouse “strain” designated *Apc*^+/*Min−FCCC*^ developed more colorectal adenomas showing an increased rate of malignant progression and rectal prolapse [80]. Importantly, the animals were housed in the same animal facility and kept on the same diet, excluding exogenous sources of the observed phenotypic differences. Nevertheless, it was shown previously that the “Western type” of diet (increased fat and reduced fiber, calcium, and vitamin D content) significantly increased the incidence of Apc-deficient intestinal tumors [81,82,83,84]. Finally, different gene variants were examined to either enhance or attenuate the *Apc^+/Min^* phenotype. More than 10 genes called modifiers of Min (*Mom*) were discovered to date. The mechanism of action of *Mom* genes was described elsewhere [85].

### 3.2. Models Producing Mutant *Apc* Variants Longer Than Apc Protein Expressed from the Apc^Min^ Allele

Although the *Apc^+/Min^* strain is a commonly used model for intestinal neoplasia, most human mutations present in sporadic or hereditary intestinal neoplasms generate a longer form of APC protein than the one expressed from the *Apc^Min^* allele. In human tumors, at least one *APC* allele produces a truncated protein retaining a functional β-catenin binding 20AAR motif [86,87]. Therefore, two mouse alleles—designated *Apc^1322T^* and *Apc^1309^* (original allele names are used throughout the review)—were generated; the alleles express the truncated Apc protein retaining one 20AAR. *Apc^+/1322T^* mice produced over 200 small intestinal polyps by the age of 10 to 12 weeks, which represented a more severe phenotype than the one observed in *Apc^+/Min^* animals. Surprisingly, although expression profiling showed that the messenger RNA (mRNA) levels of stem-cell marker leucine-rich repeat-containing G-protein coupled receptor 5 (*Lgr5*) were increased, nuclear β-catenin levels were lower than in *Apc^+/Min^* mice [88,89]. Since both strains, i.e., *Apc^+/1322T^* and *Apc^+/Min^* mice, were backcrossed with C57BL/6 animals, the discrepancy between the smaller amount of nuclear β-catenin and the more severe phenotype observed in *Apc^+/1322T^* mice cannot be explained by different genetic backgrounds. Nevertheless, the above observation can be explained by the finding that, when a certain level of nuclear β-catenin is exceeded, the production of intestinal tumors is (paradoxically) reduced [90]. In contrast, *Apc^+/1309^* mice have a milder intestinal phenotype than *Apc^+/Min^* mice, as they developed about 30 polyps by the age of 12 to 14 weeks. Moreover, the animals were affected by hyperlipidemia, a disorder characterized by abnormally elevated levels of lipids in the blood, at a younger age than *Apc^+/Min^* mice [91,92]. The difference between the pathological manifestations documented in these mouse strains is striking, as the positions of the Apc protein truncation are only 13 amino acids apart. However, it might be explained by the differences in the gene targeting strategies used to generate the animals. Unfortunately, mice harboring the *Apc^1309^* allele are not available in Europe or United States, and a detailed protocol describing the strain generation was not reported in English.

As mentioned, the C-terminus of APC is frequently lost in CRC, indicating that it is essential for the APC tumor suppressive role [93]. For functional studies of the C-terminal portion of the protein, a mouse model named *Apc^ΔSAMP^* was created. In these mice, a central region of the *Apc* gene, which encodes six β-catenin binding 20AAR motives and all AXIN-binding SAMP repeats, was deleted, while the C-terminus was retained intact. *Apc^+/ΔSAMP^* mice exhibited the same phenotype as mice harboring the *Apc^1322T^* allele, which suggested that the presence of the C-terminal part of Apc is not sufficient to suppress tumorigenesis [94]. Moreover, three additional alleles were created; the alleles were designated *Apc^1638N^*, *Apc^1638T^*, and *Apc^1572T^*. The *Apc^1638N^* allele was generated by insertion of the phosphoglycerate kinase (PGK)–neomycin selectable marker cassette into exon 15 of *Apc* in reverse orientation. The insertion should have caused a truncating mutation at codon 1638. However, truncated Apc was not detectable by Western blotting, suggesting that *Apc* mRNA translation was possibly attenuated by the anti-sense transcript generated from the neomycin expression cassette; thus, the *Apc^1638N^* allele is, in fact, a null allele [95]. While *Apc^1638N/1638N^* homozygotes died prenatally, heterozygous *Apc^+/1638N^* mice were viable and developed several (five to six) adenomas and adenocarcinomas located close to the periampullary area of the small intestine. Moreover, all *Apc^+/1638N^* mice developed cutaneous follicular cysts and desmoid tumors [96]. Therefore, *Apc^+/1638N^* mice phenocopied some of the symptoms observed in humans with the attenuated adenomatous polyposis coli (AAPC) syndrome. Hereditary AAPC is manifested by fewer than intestinal 100 polyps, delayed age of the polyp onset, and presence of severe desmoid tumors, osteosarcomas, and epidermoid cysts [97,98,99]. The *Apc^1638T^* allele was generated by insertion of the PGK–hygromycin resistance cassette into exon 15 of the *Apc* gene in the sense orientation. In this arrangement, a truncated 1638-amino-acid-long polypeptide was indeed produced from the *Apc* locus. Surprisingly, *Apc^1638T/1638T^* mice were viable and tumor-free, thus displaying a remarkably different phenotype than that observed in *Apc^1638N/1638N^* and *Apc^+/1638N^* strains. Nevertheless, when compared to wild-type (wt) mice, the small intestine of *Apc^1638T/1638T^* animals was significantly shorter, migration and proliferation of intestinal epithelial cells was faster, and the numbers of Paneth and goblet cells were increased [100]. Moreover, *Apc^1638T/1638N^* and *Apc^1638T/Min^* heterozygotes died prenatally, indicating haploinsufficiency of the *A*pc*^1638T^* allele [101]. Heterozygous *Apc^+/1572T^* animals producing the Apc protein truncated at codon 1572 were viable, but developed multifocal mammary adenocarcinomas with pulmonary metastases; homozygous *Apc^1572T/1572T^* died during embryonic development. Interestingly, in the tumor cells derived from this particular strain, a β-catenin/TCF luciferase reporter assay (TOP-FLASH) [102] and co-immunoprecipitation of β-catenin and APC indicated intermediate activation of the Wnt/β-catenin pathway. Such a level of Wnt signaling is possibly insufficient for development of intestinal neoplasia, but it might initiate breast cancer [103].

### 3.3. Models Producing Mutant *Apc* Variants Shorter Than Apc Protein Expressed from the *Apc^Min^* Allele and a Strain with Complete *Apc* Deletion

This chapter discusses seven mouse models that carry a short form of Apc, i.e., shorter than the protein expressed from the *Apc^Min^* allele. Additionally, we discuss the phenotype observed in animals after complete loss of the Apc protein, i.e., after removal of all *Apc* exons. The *Apc^Δ242^* allele was generated by inserting a *β-geo* gene trap cassette between exons 7 and 8. The targeting results in production of a fusion protein containing a truncated 242-amino-acid-long polypeptide lacking the armadillo repeat domain. *Apc^+/Δ242^* mice developed adenomas in the small intestine and colon with higher frequency than *Apc^+/Min^* mice, suggesting that the loss of the armadillo repeats increased tumorigenesis [104]. The *Apc^Δ474^* allele was created by duplication of exons 7–10 that cause a frameshift and immature stop in the *Apc* coding sequence. *Apc^+/Δ474^* heterozygotes exhibited a phenotype similar to *Apc^+/Min^* mice (polyps mainly in the small intestine and occasional mammary tumors) [33]. The *Apc^Δ716^* allele was constructed by insertion of the PGK–diphtheria toxin receptor selectable marker cassette into the *Ap*c locus. The insertion leads to expression of a truncated transcript encoding a 716-amino-acid-long Apc polypeptide. Interestingly, although the protein produced in *Apc^+/Δ716^* mice is longer than in *Apc^+/Δ242^* and *Apc^+/Δ474^* animals, the number of polyps (>400) in *Apc^+/Δ716^* mice was remarkably higher than in the first two mouse strains [105].

**Figure 3:**
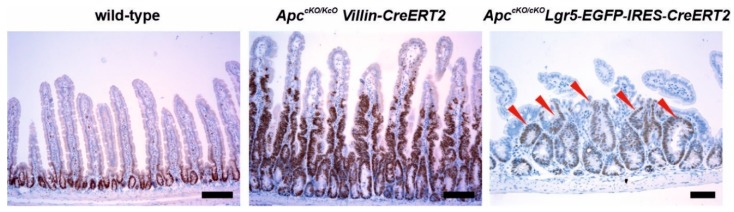
Crypt hyperplasia and microadenomas arising in the Apc-deficient small intestine. Immunohistochemical localization of proliferating cell nuclear antigen (PCNA; brown cell nuclei) in mice of the indicated genetic background. The middle microphotograph shows the hyperplastic crypt compartment developed in *Apc^cKO/cKO^ Villin-CreERT2* mice seven days after tamoxifen administration; the right image shows microadenomas (red arrowheads) formed in the *Apc^cKO/cKO^ Lgr5-EGFP-IRES-CreERT2* small intestine 21 days after tamoxifen administration. Sections were counterstained with hematoxylin (blue nuclear signal); scale bar: 0.3 mm (adopted from Reference [111]).

Three independent research groups generated mouse strains harboring conditional knock-out (cKO) alleles of the *Apc* gene with exon 14 flanked, i.e., “floxed”, by *loxP* sequences [106,107,108]. Non-recombined homozygotes of all three strains (the non-recombined alleles were termed *Apc^580S^*, *Apc^cKO^*, and *Apc^3lox14^*, respectively) were viable without any phenotype. Cre-mediated excision of exon 14 results in formation of the stop codon and production of a truncated Apc protein; Cre-recombined alleles were indicated as *Apc^580D^*, *Apc^Δ580^*, and *Apc^Δ14^*, respectively. Shibata and colleagues injected a Cre-expressing adenovirus into the lumen of the colorectal region of *Apc^580S/580S^* mice, which resulted in formation of colorectal adenomas in 80% of experimental animals [106]. To generate heterozygous animals harboring a germline knock-out *Apc* allele, *Apc^cKO^* and *Apc^3lox14/+^* mice were crossed with *EIIA-Cre*- and *MeuCre40*-expressing animals, respectively. In *EIIA-Cre* transgenic mice, Cre is expressed in the preimplantation embryo from early adenoviral (*EIIA*) promoter active in all tissues; in MeuCre40 mice, the Cre recombinase is expressed in all tissues. Animals from both strains developed numerous intestinal tumors, and subsequent analysis indicated that the wt *Apc* allele was inactivated by allelic loss [34,107]. Moreover, tamoxifen-induced recombination of the *Apc^cKO^* alleles in *Apc^cKO/cKO^ Lgr5-EGFP-IRES-CreERT2* and *Apc^cKO/cKO^ Villin-CreERT2* animals allowed tissue-specific Apc inactivation in intestinal stem cells or in all intestinal epithelium cells, respectively [109,110]. In the latter strains, massive crypt hyperproliferation followed by intestinal microadenoma formation was observed already several days after tamoxifen administration (Figure 3) [111].

In addition to the mouse strains harboring floxed exon 14, Robanus-Maandag and colleagues generated a strain with floxed exon 15 (*Apc^15lox^*). Deletion of this particular exon in germ cells generated *Apc^+/Δ15^* mice that displayed a phenotype reminding of *Apc^+/Min^* mice. Additionally, the *Apc^+/15lox^* mice were crossed to transgenic mice expressing Cre recombinase from the fatty acid-binding protein (*Fabpl*) gene promoter; the promoter is active in epithelial cells of the distal small intestine and colon. These mice survived longer (than *Apc^+/Δ15^*) and developed about 40 tumors in the ileum, colon, and rectum [112].

Whereas the majority of human colorectal tumors harbor truncated *APC*, the null variant of the *APC* gene is relatively uncommon. In order to study the effect of complete loss of APC, Cheung and colleagues produced a mouse strain harboring cKO alleles allowing deletion of all 15 *Apc* exons (the recombined allele was designated *Apc^Δe1–15^*). *Apc^+/Δe1–15^* heterozygotes had a more severe intestinal phenotype than *Apc^+/Min^* mice. Importantly, as the wt *Apc* allele was inactivated in *Apc^+/Δe1–15^* animals by *Apc* promoter hypermethylation or loss of heterozygosity, it was evident that, in the mouse model, the presence of a truncated Apc protein is not required for intestinal tumor development. Interestingly, although the amount of β-catenin protein was similar in tumors of *Apc^+/Δe1–15^* and *Apc^+/Min^* mice, the levels of β-catenin-dependent transcription seemed to be lower in *Apc^+/Δe1–15^* animals [113]. This confirmed that the “just optimal” β-catenin level is necessary for tumor initiation and growth [90].

### 3.4. Models Expressing Stabilized β-Catenin

Although *APC* mutations initiate the majority of human CRCs, a subset of human colorectal tumors with intact *APC* carries protein-stabilizing mutations in *CTNNB1*. For β-catenin ubiquitination and subsequent proteasomal degradation, the conserved N-terminal serine and threonine residues (S33, S37, T41, and S45) have to be phosphorylated. These amino acids are encoded by exon 3 of the *CTNNB1* gene; the same exon is considered to be a mutation hotspot in human CRC. Missense mutations or short deletion affecting the critical amino-acid residues (the mutational changes preserve the open reading frame) prevent β-catenin phosphorylation and, thus, lead to production of a stable protein (reviewed in References [114,115]). In order to model tumors that are initiated by alterations in the *CTNNB1* gene, Harada and colleagues generated mice harboring a conditional *Ctnnb1* allele where exon 3 was flanked by *loxP* sites (*Ctnnb1^lox(ex3)/lox(ex3^*^)^). These mice were crossed with knock-in mice expressing Cre recombinase under the control of the cytokeratin 19 promoter (*Krt1–19^Cre^*); the promoter drives Cre expression in the intestinal epithelium starting at early embryonic stages. Heterozygous *Ctnnb1^+/lox(ex3)^ Krt1–19^+/Cre^* animals developed over 3000 polyps in the duodenum and proximal jejunum and only microadenomas in the colon by the third week after birth. Alternatively, *Ctnnb1^lox(ex3)/lox(ex3^*^)^ mice were crossed with the *Fabpl^Cre^* strain; heterozygous *Ctnnb1^+/lox(ex3)^* *Fabpl^Cre^* animals developed 200 to 700 polyps in the small intestine by the age of 4–5 weeks [116]. In summary, the models of β-catenin oncogenic activation recapitulated a severe phenotype observed in some Apc-deficient mice.

### 3.5. Alleles Allowing Aberrant (Over) Expression of Wnt Agonists R-Spondins

Secreted RSPOs bind the Lgr 4/5/6 receptor to potentiate the Wnt signaling pathway output. The signaling function of the RSPO/LGR complex has multiple effects and, inter alia, leads to inhibition of transmembrane E3 ubiquitin ligases zinc and ring finger 3 (ZNRF3) and ring finger 43 (RNF43). These ligases act on Wnt receptor Frizzled, mediating its turnover. However, binding of the RSPO ligand to the LGR receptor results in ZNRF3 and RNF3 internalization and subsequent degradation in lysosomes. The mechanism leads to increased availability of the Frizzled receptors on the cell surface and, thus, enhanced Wnt signaling (Figure 1b) (reviewed in Reference [117]). 

Approximately 10% of CRC specimens harbor chromosomal rearrangements that involve loci encoding *RSPO* genes. These chromosomal rearrangements are mainly based on gene fusions of *RSPO2* or *RSPO3* with another highly expressed gene, such as protein tyrosine phosphatase receptor type K (*PTPRK*), eukaryotic translation initiation factors 3e (*EIF3E*), and piezo-type mechanosensitive ion channel component 1 (*PIEZO1*) [118,119]. All these gene fusions result in aberrant *RSPO2/3* overexpression. To investigate this type of CRC, Hilkens and colleagues developed a conditional *Rspo3* transgenic mouse (*Rspo3^inv^*) where *Rspo3* was expressed in cells producing Cre recombinase. The mice were crossed to *Lgr5-EGFP-IRES-CreERT2* [110] mice, and Cre-mediated *Rspo* expression was induced by tamoxifen. The animals developed hyperplasia in the small intestine, cecum, and proximal colon. The incidence of neoplasia (mainly adenoma and adenocarcinoma) was 2.5 tumors per mouse on average, and moderate upregulation of Wnt target genes was observed [120]. Additional mouse models were generated by Cas9-mediated fusion of *Rspo2* or *Rspo3* with *EIF3E* and *Ptprk*, respectively, using the tetracycline-inducible clustered regularly interspaced short palindromic repeats (CRISPR)/Cas9 system. Since the chromosomal rearrangements occurred randomly after the Cas9-mediated DNA cleavage, this model adequately reproduced the condition that is commonly found in human CRC. Two weeks after doxycycline administration, i.e., after Cas9 induction, adenomas were observed in the mouse small intestine. Nevertheless, in both models, tumor growth was rather attenuated, and hyperplastic or dysplastic lesions were formed only. Surprisingly, contrary to the model of Hilkens and co-workers, no significant increase in Wnt target gene expression in the *EIF3E-Rspo2* or *Ptprk-Rspo3* intestines was noted [121].

## 4. Mouse Models of Inactive Hippo Signaling

The Hippo signaling pathway was originally discovered in *Drosophila* as a signaling mechanism controlling the organ size. However, later studies identified involvement of the Hippo signaling in other important processes such as cell division, differentiation, and maintenance of cell pluripotency [122]. The core complex of the mammalian Hippo signaling pathway includes serine/threonine STE20-like protein kinase 1 (MST1; alternative name STK4) and related MST2 (STK3), large tumor suppressor kinase 1/2 (LATS1/2), scaffold proteins salvador family WW domain-containing protein 1 (SAV1), and mono-polar spindle-1 one binder kinase activator 1A/1B (MOB1A/1B). When the Hippo pathway is not active, the effectors yes-associated protein 1 (YAP1) and tafazzin (TAZ) can freely enter the cell nucleus, where they associate with transcription co-factors from the transcriptional enhancer factor 1 and abacus A family (TEAD). The YAP1 (TAZ)–TEAD complex activates transcription of pro-proliferative and anti-apoptotic genes. Conversely, when Hippo signaling is activated (by growth inhibiting signals), YAP and TAZ are phosphorylated by LATS1/2. The modification prevents their transport to the nucleus and drives their ubiquitination and degradation (reviewed in Reference [123]). The pathway is further controlled by ubiquitination-independent proteasome activator subunit 3 (PSME3, alternative name regenerating islet-derived protein 3 (REGγ)), which can degrade LATS1 and, thus, activate YAP1. 

Neither deregulation of the Hippo pathway nor mutations in genes encoding the pathway components were reported in relation to a particular CMS group. Nevertheless, some CRCs show a positive correlation between poorer prognosis and overexpression of *YAP1*, *TAZ*, *TEAD4*, and *REGγ* [124,125,126,127,128]. In addition, YAP1 and TAZ proteins interact with β-catenin. The interaction leads to inhibition of β-catenin nuclear localization and results in downregulation of Wnt signaling. Moreover, since active Hippo signaling inhibits cells growth and proliferation, the signaling in fact opposes pro-proliferative Wnt pathway-mediated cellular processes. Consequently, models altering the Hippo pathway status might complement studies involving aberrant Wnt signaling.

The first model simulating the inactive Hippo pathway was represented by a transgenic mouse strain allowing doxycycline-inducible *Yap1* production/activation. Upon doxycycline administration, the mice ubiquitously expressed a mutated form of Yap1 (Yap1^S127A^), which is not phosphorylated on critical serine 127 and, thus, escapes degradation. The mice (examined five days after activation of Yap1 expression) displayed massive cell proliferation in multiple organs. The most pronounced phenotype was observed in the intestine, where the entire epithelium appeared dysplastic. Interestingly, the proliferation was not restricted to the intestinal crypts, but dividing cells were also detected in the villus region. In addition, mature goblet or Paneth cells were absent throughout the intestine [129]. Additionally, the same research group generated a mouse strain with Yap1^S127A^ expression regulated by intestinal epithelium-specific expression of reverse tetracycline transactivator (rtTA). Interestingly, the phenotype of these mice was fundamentally different from the animals expressing Yap1^S127A^ ubiquitously. Strikingly, seven days after induction of Yap1^S127A^, the intestinal epithelium exhibited progressive degeneration associated with loss of dividing cells in the crypts [130]. It was suggested that, in the whole-body Yap1 activation model, paracrine Yap1-dependent signals originated from stromal cells might support adjacent epithelial cells, and this type of support is absent in animals with tissue-specific Yap1^S127A^ expression [123]. Nevertheless, the discrepancy between the phenotypes observed in the above-described models remains unclear. Another model of the inactive Hippo pathway was based on null alleles of the *Mst1* gene (*Mst1^null^*) and conditional *Mst2* alleles (*Mst2^ff^*); to achieve epithelial inactivation of Mst2, the latter strain was intercrossed with transgenic *Villin-Cre* mice [109]. The *Mst1^null^* or *Mst2^ff^ Villin-Cre* mice were born in Mendelian ratios, but their average lifespan was 13 weeks only. The mice displayed a significantly expanded stem-cell compartment and reduced numbers of differentiated cells in both small intestine and colon; moreover, adenomas were present in the distal part of the colon. Interestingly, whereas the total amount of β-catenin was not—in comparison to control wt mice—changed, the level of nuclear β-catenin was increased. Additionally, the phenotype of *Mst1^null^ Mst2^ff^ Villin-Cre* mice was suppressed after inactivation of one or both *Yap1* alleles [131]. Rather surprisingly, inactivation of *Yap1* per se in the intestine had no obvious phenotype. However, when subjected to DSS treatment, the regenerative capacity of the Yap1-deficient intestinal epithelium animals was abolished [132]. Enhanced Hippo signaling was also investigated in *REGγ^−/−^* mice. REGγ deficiency increased expression of Lats1, and, as a consequence, the cellular level of phosphorylated Yap1 was upregulated. Nevertheless, after DSS-induced colitis, *REGγ^−/−^* mice developed lower amounts of smaller and less proliferating colorectal tumors when compared to wt mice [128]. In summary, the described models (the corresponding strains are listed in Table S3, Supplementary Materials) indicated that impaired Hippo signaling via Yap1 is involved in intestinal tumorigenesis. 

## 5. Mouse Models of p53 Pathway Deficiency

Activation of tumor suppressor p53 represents a fundamental mechanism blocking cancer cell proliferation and/or survival. Consequently, p53 loss is associated with initiation, progression, and invasiveness of various malignancies (reviewed in Reference [133]). In a healthy cell, the p53 level is kept low by action of E3 ubiquitin ligase mouse double minute 2 homolog (MDM2) [134]. Nevertheless, when the cell is exposed to adverse conditions such as oxidative stress, DNA damage, or replication stress, p53 is stabilized and induces apoptotic pathways (reviewed in Reference [135]). Moreover, to block cell-cycle progression, p53 activates transcription of many target genes involved in cell-cycle regulation. A prototypic p53-induced gene is represented by cyclin-dependent kinase inhibitor 1A (*CDKN1A*), which encodes cyclin-dependent kinase (CDK) inhibitor p21 (alternative name CIP1/WAF1); p21 prevents cells from entering the synthesis (S) phase (reviewed in Reference [136]). Loss of the p53 function was detected in 50–70% of all colorectal tumors [16,137]; nevertheless, p53 mutations were mostly detected in advanced tumors. Thus, p53 inactivation represents one of the crucial events in adenoma-carcinoma transition. Moreover, *TP53*-mutant tumors appear to be more resistant to chemotherapy, resulting in poorer prognosis of the treated patient (reviewed in Reference [24]). Mutations in the *TP53* gene were found in tumors of all CMS types, ranging from 27% to 62% in the CMS1 or CMS2 group, respectively [24]. The most frequently mutated region of the *TP53* gene consisted of exons 5–8 that encode a sequence-specific DNA-binding domain. Intriguingly, mutations in codons 175, 245, 248, 273, and 282 were repeatedly identified in several studies [35,138,139]. These predominantly missense mutations affect the p53 ability to bind target DNA, and consequently they inhibit the transcriptional regulatory role of p53. Interestingly, different *TP53* mutations might impact CRC properties, especially lymphatic or vascular invasion and metastasis (reviewed in Reference [140]). Inactivation of the p53 target gene *CDKN1A* was detected in 79% of colorectal tumors, and it showed a clear correlation with *TP53* deficiency [141]. Strikingly, p21 loss inversely correlated with high CIMP and MSI. Moreover, in CIMP- and MSI-high CRCs, the deficiency was independent of the *TP53* status [142]. Therefore, colorectal tumors with mutated p21 were assigned to the CMS2 or CMS4 groups that display low CIMP and MSI and contain a high proportion of p53-mutated tumors [24].

A whole-body knockout of the *Trp53* gene in the mouse was described more than 25 years ago. The study confirmed the tumor suppressive role of p53; p53-deficient mice were predisposed to formation of many different types of tumors, predominantly lymphomas, osteosarcomas, and adenocarcinomas [143,144]. Combinations of p53 deficiency with other mouse tumor models modulated the rate, localization, and number of gastrointestinal tumors. For example, *Apc^+/Min^ Trp53^−/−^* mice developed increased amounts of more invasive intestinal adenomas than *Apc^+/Min^* mice harboring wt p53 [145]. In addition, *Trp53^−/−^ Tcrβ^−/−^* mice suffered from more severe colitis than *Tcrβ^−/−^* mice and developed inflammation-associated adenocarcinomas in the cecum and colon [146]. Interestingly, AOM/DSS treatment in p53-deficient mice resulted in nuclear accumulation of β-catenin accompanied by robust activation of Wnt-responsive genes. However, increased Wnt/β-catenin-dependent transcription was not seen when animals were treated with DSS only [147].

Interestingly, in the case of CAC, p53 deficiency influenced not only the incidence, but also the morphology of the tumors. Comparison of tumors isolated from DSS-treated *Trp53^−/−^*, *Trp53^+/−^,* and *Trp53^+/+^* mice showed that *Trp53^−/−^* tumors are rather flat (84.6%), while *Trp53^+/−^* and *Trp53^+/+^* lesions are mostly polypoid (83.3% and 100%, respectively; polypoid tumors represent neoplastic lesions whose height is greater than one-half of their diameter). Moreover, polypoid neoplasia often carried (in 75% of cases) mutations in the *Ctnnb1* gene, and tumor cells displayed nuclear localization of β-catenin. The results suggest that different tumorigenic mechanisms affect not only the formation, but also the morphology of CAC [148].

In addition to the FAP syndrome, there are several other hereditary polyposis syndromes including the Peutz–Jeghers syndrome (PJS). Individuals with PJS develop gastrointestinal hamartomatous polyps due to an inactivating germline mutation in the liver kinase B1 (*LKB1*) gene (alternative name serine/threonine kinase 11 (*STK11*)). In contrast to the polyps developed in FAP patients, malignant transformation of PJS hamartomas is very rare (reviewed in Reference [149]). LKB1 physically associates with p53 and promotes p53-dependent apoptosis [150]. Importantly, restoration of LKB1 activity in (originally) LKB1-defective cancer cells induced p21 expression followed by cell-cycle arrest [151,152]. In order to investigate the LKB1 function in PJS, mice harboring mutation in the *Lkb1* gene were generated. Homozygous germline deletion of *Lkb1* was embryonic lethal; however, heterozygous mice developed hamartomatous gastric and small intestinal polyps [153]. In addition, *Lkb1^+/−^ Trp53^−/−^* mice displayed increased incidence and earlier formation of tumors that retained a hamartomatous character [154], indicating that combined deficiency in both genes might accelerate tumor formation.

As already indicated, CDK inhibitor p21 (*Cdkn1a*) is important regulatory protein involved in cell proliferation. Surprisingly, although *p53^−/−^* mice develop multiple tumors, spontaneous tumor development was not observed in young *Cdkn1a^−/−^* mice [155,156]. However, when the mice were reared for one year or longer, formation of hematopoietic, endothelial, and epithelial tumors was noted [157]. Importantly, *Cdkn1a*-deficient mice developed increased numbers of ACFs along the entire length of the colon after treatment with AOM [158]. Moreover, tumor incidence and metastatic potential was further potentiated by whole-body irradiation [159]. Similarly to *Trp53^−/−^ Apc^+/Min^* mice, the increased tumor burden was observed in *Cdkn1a^−/−^ Apc^+/1638^* animals [160]. The results suggested that the p53–p21 pathway plays an important role in the inhibition of growth of Apc-deficient tumors. Indeed, in human tumors, p21 loss indicates poor prognosis [161]. In conclusion, mutations inactivating p53 were manifested by increased incidence of neoplasia in other organs than the intestine. Therefore, to model CRC, p53 pathway-deficient mice were mainly employed in combination with other genetic modifications (or with irradiation and mutagen exposure) to provoke (or accelerate) intestinal tumor development and progression. Models described in this chapter are listed in Table S4 (Supplementary Materials).

## 6. Mouse Models of Aberrant Activation of the Epidermal Growth Factor Signaling Pathway

The signaling pathway initiated by interaction of the epidermal growth factor (EGF) ligand and the EGF receptor [(EGFR; alternative names avian erythroblastic leukemia viral (v-erb-b) oncogene homolog (ERBB1) or human epidermal growth factor receptor 2 (HER1)] represents a signaling cascade inducing pleiotropic effects in the target cell. The effects include proliferation and inhibition of apoptosis; therefore, the pathway activity is tightly regulated (reviewed in Reference [162]). Ligand binding to EGFR triggers sequential activation of mitogen-activated protein kinases (MAPKs), which transduces the signal to the cell nucleus. In more detail, EGFR functions as a transmembrane receptor tyrosine kinase that undergoes autophosphorylation upon interaction with EGF. A phosphorylated intracellular portion of the receptor interacts with the Src homology 2 (SH2) domain of the cytoplasmic proteins growth factor receptor-bound protein 2 (GRB2) and son of sevenless (SOS). Receptor complex-bound SOS promotes the exchange of guanosine diphosphate (GDP) to guanosine triphosphate (GTP) associated with small G-proteins from the RAS family. GTP-loaded Ras proteins in turn activate Raf protein kinases, the initial kinases in the MAPK cascade (reviewed in Reference [162]).

In human sporadic CRC, several principal components of the MAPK pathway, i.e., *EGFR*, *KRAS*, *NRAS*, and *BRAF*, are recurrently mutated. Generally, activating mutations in proto-oncogenes *KRAS* and *BRAF* were present in human tumors corresponding to the CMS3 (68%) and CMS1 (42%) groups, respectively. Whereas *BRAF* mutations were almost exclusively present in these CMS groups, *KRAS* mutations were also detected, although to a lesser extent, in the CMS2 and CMS4 groups. Interestingly, in tumor-derived intestinal organoid cultures, *KRAS* mutations were found in all CMS groups except for CMS3 [26]. Mutations in *NRAS* were mostly detected in the CMS3 group (9%) [24]. 

To analyze the impact of genetic alterations in the EGFR pathway on CRC initiation or progression, a number of mouse models were used. According to mouse studies, mutations in the EGFR pathway alone are not sufficient to initiate colon tissue transformation [163,164]. Nevertheless, oncogenic mutations in genes involved in EGFR-mediated signaling are considered to be driver mutations as they emerge in early (pre-neoplastic) lesions. In fact, activating mutations in *KRAS* and *BRAF* were already detected in tumor-initiating cells [165,166]. Additionally, when these genetic alterations are combined with mutations in genes encoding *Trp53* or Wnt pathway components, they facilitate colorectal tumor progression. 

### 6.1. Mouse Strains Expressing Mutant Epidermal Growth Factor Receptor

Activating mutations in the *EGFR* gene were found in 10% of the analyzed human tumor specimens. Moreover, 7% of CRCs harbored activating mutations in EGFR paralog ERBB2/HER2 [167]. The EGFR function in CRC was assessed using mice carrying the *Egfr^wa2^* [168] and *Egfr^wa5^* [169] loss-of-function alleles, and *Egfr^tm1Mag^* [170] null allele using various genetic backgrounds. Whereas *EGFR* gene amplification and activating mutations in the receptor kinase domain are frequent in human CRC samples [171,172], experiments in mice showed that the EGFR activity is indispensable for tumors developed in *Apc^+/Min^* mice [170] or in AOM/DSS-induced neoplasia [168]. To assess the Egfr function in immune-mediated colitis, *Egfr^wa5/wa5^* mice were treated with AOM/DSS and crossed with interleukin 10 (Il10)-deficient (*Il10^−/−^*) mice, a strain that represents a model of spontaneous colitis with many characteristics of human inflammatory bowel disease (IBD). Although the incidence of tumors in AOM/DSS-treated *Egfr^wa5/wa5^* mice was comparable to wt controls, tumor progression was significantly increased. In 40% of AOM/DSS-treated *Egfr^wa5/wa5^* mice, invasive adenocarcinomas were formed; tumors in wt mice remained non-invasive. In contrast, *Il10^−/−^ Egfr^wa5/wa5^* mice exhibited elevated tumor formation and progression in comparison to *Il10^−/−^ Egfr^+/+^* mice. Since the tumors in *Il10^−/−^ Egfr^wa5/wa5^* animals developed without administration of (any) mutagen, this model might be more applicable to studying tumorigenesis in IBD patients. Nevertheless, the results of these experiments paradoxically indicated an unexpected tumor-suppressive function of EGFR signaling in chronic colitis [169].

### 6.2. Mouse Models Producing Mutant *Kras* and *Nras*

*KRAS* mutations that “lock” the protein in the active GTP-bound state were detected in approximately 40% of human CRCs [173]. Mutations in the homologous *NRAS* gene were identified in less than 5% of sporadic CRCs. As *KRAS* is the most frequently mutated oncogene participating in EGFR signaling in human CRC, great effort was made to characterize the *KRAS* function using animal models. In human tumors, activating *KRAS* mutations are localized to the region that encodes the GTP-binding domain, specifically to codons 12 and 13. Accordingly, mouse alleles harboring substitutions in amino-acid glycine at position 12 or 13 (G12 and G13) were used to model colorectal carcinogenesis. In general, phenotypical and histological analyses of the *Kras*-mutant colonic epithelium converged on the fact that the *Kras* oncogene enhanced proliferation but was insufficient for cell transformation. However, in combination with other driver mutations, such as in *Apc* or *Trp53*, mutant *Kras* indeed promoted tumor progression [164,174]. Additionally, several research groups generated mouse strains carrying *Kras* alleles with inducible substitution of the glycine 12 residue to aspartate (G12D) or valine (D12V). Johnson and colleagues prepared two “latent” alleles (*Kras^LA1^* and *Kras^LA2^*), which were activated by spontaneous (mutual) recombination of wt and oncogenic *Kras^G12D^* variant of exon 1. The *Kras^LA1^* allele contains only one copy of the mutated exon 1, while the *Kras^LA2^* allele contains two copies. Thus, in vivo recombination of the *Kras^LA1^* allele produces both wt and *Kras^G12D^* allele (in a 1:1 ratio), whereas the *Kras^LA2^* allele generates the oncogenic *Kras^G12D^* allele only. The frequency of recombination ranged from 10^−3^ to 10^−7^ per cell generation, which (surpassingly) ensured sufficient cell numbers expressing mutant Kras. Mice harboring the latent allele developed colonic aberrant crypt foci (ACF), which represent pre-neoplastic epithelial lesions with enhanced proliferation and potential for malignant growth [165]. However, ACF found in *Kras^LA1^* and *Kras^LA2^* mice did not progress to form more advanced tumors. This suggested that *Kras* was not sufficient for malignant transformation of epithelial cells [164]. Interestingly, the presence of *Kras^LA1^* and *Kras^LA2^* alleles on the *Apc^+/Min^* and *Trp53^−/−^* genetic background had—presumably due to the low incidence of oncogenic *Kras* allele activation—no or little effect on ACF progression, The only detectable effect was occurrence of several adenocarcinomas in the duodenum [164].

In order to maximize the effect of oncogenic *Kras*, additional alleles were designed. Guerra and colleagues generated mice harboring the conditional *Kras^G12V^-IRES-β-geo* allele and crossed the animals with mice that expressed tamoxifen-inducible Cre-ERT2 recombinase from the promoter of the large subunit of RNA polymerase II (*RERTn*); the allele produced upon Cre-mediated recombination was named *Kras^V12^*. Since the homozygous *Kras^V12/V12^* animals died during embryonic development, heterozygous *Kras^+/V12^ RERTn^+/ERT^* mice were utilized in further experiments. However, these mice did not reveal any pathologic changes in the intestinal epithelium [175]. In contrast, the similar inducible *Kras^G12D^* allele, which was specifically activated in the intestinal epithelium, caused hyperproliferation of cells in the colon crypts of *Kras^G12D^ Fabpl^cre^* mice [163,174]. Moreover, the oncogenic form of *Kras* in the colon of Apc-deficient mice (*Apc^2lox14/+^ Kras^G12D/+^ Fapbl-Cre* strain) markedly increased the number of tumors, and, by blocking cell differentiation, Kras^V12^ induced tumor progression [174]. Interestingly, the *Nras^G12D^* allele in the analogous genetic background neither enhanced proliferation of the healthy colonic epithelia nor promoted progression of Apc-deficient adenomas. However, the mutant *Nras^G12D^* allele had the capacity to suppress DSS-mediated apoptosis in the colonic epithelium [174]. Finally, mice harboring the *Kras^G12D^-IRES-EGFP* allele (the allele was designated *Kras^Asp12^*) were crossed with *Ah-Cre* mice that produce Cre in various tissues after induction with β-naphthoflavone [176]. The *Kras^Asp12^ Ah-Cre* mice developed several adenomas in the small intestine and colon within two years after Cre induction. However, after crossing with *Apc^+/Min^* mice and Cre induction with β-naphthoflavone, the compound mutants (*Kras^Asp12^ Ah-Cre Apc^+/Min^*) displayed a much severer phenotype than *Apc^+/Min^* mice, i.e., decreased lifespan and elevated amounts of small intestinal and colonic tumors [177]. As an alternative approach to Cre-expressing mouse strains, Hung and colleagues accelerated colon adenocarcinoma progression by injection of adenoviral Cre into the colon of *Apc^cKO/cKO^* *Kras^+/G12D^* mice [178]. Most recently, a novel *Kras^A146T^* allele that mimics less frequent mutation in the Kras guanine nucleotide-binding domain found in human CRC was established and expressed after *Fabp1^cre^*-mediated recombination in the colon of wt and *Apc^2lox14/+^* mice. However, the effect of the mutated protein on the intestinal epithelium was milder when compared to the phenotype observed in animals expressing the *Kras^G12D^* allele [179].

### 6.3. Mouse Models Harboring Mutant *Braf* Alleles 

Another recurrently mutated gene in the EGFR pathway that was genetically manipulated in mice is *BRAF* [180]. The *BRAF* gene was mutated in approximately 10% of colorectal adenocarcinomas [37]. The majority of *BRAF* mutations in human cancers are localized to the region encoding a kinase domain; the gene alterations mainly result in amino-acid substitution from valine (V) to glutamic acid (G) in codon 600 (V600E missense mutation; the mutation was formerly known as V599E) [180,181]. To study the function of aberrantly activated *BRAF* in tumorigenesis, Mercer and colleagues generated the mouse allele *Braf^V600E^* that allows Cre-inducible expression of the oncogenic *Braf* variant [36]. Shortly after the study was published, Dankort and colleagues produced a similar Cre-inducible *Braf^V600E^* allele and used the allele to analyze the *Braf* function in lung adenocarcinomas [182]. Unfortunately, none of these mouse models were employed to study colon tumorigenesis. Finally, in 2013, intestine-specific recombination of the third version of the *Braf^V600E^* allele was carried out by cross-breeding of *Braf^+/V600^* mice with the *Villin-Cre* strain. Expression of the *Braf^V600E^* oncogene in the mouse intestinal epithelium resulted in crypt hyperplasia with a high rate of tumor progression. Although the presence of the *Braf^V600^* allele was sufficient to transform cells, gene expression and immunohistochemical analysis of advanced tumors showed that additional mutations in genes encoding the Wnt and p53 pathways components were required for tumor progression [183]. Additionally, organoids derived from the *Braf^V600E^* mouse were employed in experiments (the allele activation was achieved by infection of organoid cells with Cre-expressing lentivirus) showing that age-related epigenetic changes are an important oncogenic driver in intestinal cells expressing mutant *Braf* [184]. 

Interestingly, CIMP- and MSI-high tumors, which fall to the CMS1 group of CRC with mutations in *BRAF*, often exhibit significant mucinous cell differentiation [29,185]. Moreover, a correlation between enhanced expression of mucins and the presence of somatic *BRAF^V600E^* mutation was reported recently [186]. Major glycoprotein secreted by intestinal goblet cells Mucin-2 functions as an important homeostasis-preserving protein involved in formation of the mucinous layer protecting the intestinal epithelium [187]. The protective role of Mucin-2 against tissue damage was documented in *Muc2^−/−^* mice that developed adenomas in the small intestine, colon, and rectum [188]. Since mucinous tumors frequently display poorer prognosis, we might speculate that elevated mucin expression results in increased tumor resistance towards treatment. 

In addition to MAPK signaling, the EGFR pathway activates the phosphoinositide 3-kinase (PI3K)/protein kinase B (PKB/AKT)/mammalian target of rapamycin (mTOR) signaling cascade (reviewed in Reference [162]). Mutations of critical components involved in PI3K-mediated signaling, i.e., in *PIK3*, phosphatase and tensin homolog (*PTEN*; the gene encodes a dual-specificity phosphatase that antagonizes PI3K signaling [189]), and *AKT* occurred in 13–32%, 14%, and 1–6% of human CRC samples, respectively [190,191]. Recently, Mitchell and Phillips reviewed the mouse models of mutant PI3K in disease, covering CRC in detail [192]. In addition, mouse models mimicking mutations in the *Pten* and *Akt* genes were described elsewhere [193,194,195,196]. Thus, for the sake of brevity, we do not discuss the mouse models of aberrant EGF signaling that include alterations in the *Pik3*, *PTEN*, and *AKT* genes; the mouse strains that are mentioned in this chapter are listed in Table S5 (Supplementary Materials).

## 7. Mouse Models of Impaired TGFβ Signaling

The TGFβ signaling pathway is indispensable for intestinal homeostasis as it inhibits proliferation and supports differentiation of intestinal epithelial cells. Hence, the pathway represents an important tumor-suppressive mechanism. Therefore, TGFβ signaling is often altered in sporadic CRC (reviewed in Reference [197]). In brief, TGFβ ligands exist in three isoforms (TGFβ1/2/3) and form active homo- or heterodimers. The ligand dimers bind to TGFβ receptors type II (TGFβ-RII) that subsequently recruit and phosphorylate the TGFβ-RI receptors. In the cytoplasm, phosphorylated TGFβ-RI further bind receptor-regulated SMAD signal transducers (R-SMADs), which upon phosphorylation bind the common partner SMAD4. The R-SMAD/SMAD4 complexes then shuttle into the nucleus, where they interact with a variety of transcriptional factors and regulate gene expression (reviewed in Reference [198]).

The most common mutations of the TGFβ pathway in CRC are in the *TGFBR2* gene encoding the type II receptor (nearly 30% of CRCs). Since the *TGFBR2* gene contains a microsatellite sequence in its coding region, mutated *TGFBR2* was found in more than 80% of MSI-high tumors (reviewed in Reference [199]). Mutations in individual *SMAD* genes are present in approximately 10% of CRC and predict—due to their association with disease progression and lymph node metastasis—poor prognosis [200]. Mutations in *SMAD4* are the most frequent and are associated with mucinous tumor histology [201]. Increasing incidence of *SMAD4* mutations in advanced malignancies also suggests that this transcription co-factor is involved in tumor progression [202,203]. In addition, hereditary germline *SMAD4* mutations are associated with the juvenile polyposis syndrome characterized by increased incidence of hamartomatous intestinal polyps that gradually progress to carcinomas [204]. Mutations in *SMAD2* and *SMAD3* are less frequent than in *SMAD4*, although they are very similar with respect to the mutation type and distribution in the gene region [201].

Intriguingly, the CMS4 group tumors indicated the gene expression signature of active TGFβ signaling [24]. Similarly, gene set enrichment analysis of CRC cell lines and tumor-derived organoids revealed increased activation of the TGFβ pathway in the CMS4 group cell lines and organoids. Recent studies identified cancer-associated fibroblasts (CAFs) present in the tumor stroma as a “source” of the gene expression signature, indicating elevated TGFβ signaling [205,206]. Importantly, active TGFβ signaling in the tumor microenvironment increases the count of tumor-initiating cells in the tumor [205]. Moreover, tumors enriched in TGFβ-specific transcription tend to form metastases, resulting in poor prognosis [207]. Interestingly, mutations in the TGFβ pathway are less frequent in commercially available cell lines and tumor organoids than expected from the analysis of human tumor specimens [26]. This is consistent with the fact that the tumor stroma is primarily responsible for the TGFβ signaling gene signature.

In accordance with the fact that the TGFβ pathway is involved in the immune response regulation, *Tgfb1*^−/−^ mice displayed extensive inflammation and died within one month after birth [208,209]. However, cross-breeding of *Tgfb1*^−/−^ mice with immunodeficient *Rag2*^−/−^ mice generated viable animals that developed tumors in the cecum and colon [210]. Homozygous knock-out of the *Smad2* and *Smad4* genes was embryonic lethal; however, deletion of one *Smad4* allele only yielded gastrointestinal hamartomas in the stomach and duodenum with histopathological features reminding of JPS [211]. In contrast, *Smad3* homozygous deletion did not affect embryogenesis; however, *S*mad3-deficient mice developed invasive colorectal tumors that metastasized to the lymph nodes [212].

Colorectal tumors arising as a result of impaired TGFβ signaling did not display elevated Wnt signaling [213]. This mirrored the fact that upregulation of the TGFβ and Wnt signaling pathways was observed in different CMS groups (CMS4 vs. CMS2 group, respectively) [24]. Nevertheless, deficiency in *Tgfbr1*/2 or *Smad3*/*4* further accelerated intestinal tumor development and increased malignancy of lesions formed in the Apc-deficient intestine [214,215,216,217,218]. Analogously, compound heterozygous disruption of the *Apc* and *Smad2* genes enhanced tumor progression and invasiveness [219]. Interestingly, mice with conditional *Tgfbr2* knock-out in the intestinal epithelium (*Tgfbr2^E2flox/E2flox^ Villin-CreERT2*) displayed impaired mucosal regeneration after irradiation and, moreover, developed invasive carcinomas in the colon upon colitis-inducing DSS treatment. Thus, the genetic alteration of the TGFβ pathway appears to be sufficient to generate CAC in the inflammatory microenvironment without any need for Apc inactivation [30]. Mouse strains described in this chapter are listed in Table S6 (Supplementary Materials).

## 8. Mouse Models of DNA Mismatch Repair Deficiency

The mismatch repair (MMR) mechanism provides corrections of base–base mismatches and loops in DNA strands that originate from incorrect base insertions (or deletions) during DNA replication. Nucleotide selectivity and polymerase proofreading result in the error rate of approximately 10^−5^ to 10^−6^ mismatches during DNA replication. Importantly, the functional MMR system further decreases the error rates to as low as 10^−10^ [220]. The canonical MMR pathway in humans consists of two major functional components having names derived from homologous bacterial genes, mutator S (*MutS*) and mutator L (*MutL*). MutS contains the MutS homolog 2 (MSH2) protein, which forms a heterodimer with the MSH6 protein, in the case of base substitutions and small loop repairs, or with MSH3, in the case of larger DNA loops. Heterodimer MutL, formed with MutL homolog 1 (MLH1) in combination with postmeiotic segregation increased 1/2 (PMS1/2) or MLH3, is involved in the recognition and repair of non-Watson–Crick base pairs. MMR deficiency leads to a higher mutation rate and occurs in cancers with MSI. Thus, intestinal tumors with mutations in the MMR pathway genes were assigned to the CMS1 group. As might be expected, the increased presence of neoantigens generated as a result of non-functional MMR also leads to significant infiltration of the CMS1 group tumors by immune cells. Impaired MMR is also associated with hereditary nonpolyposis colorectal cancer, so-called Lynch syndrome. Moreover, increased MSI was found in patients with ulcerative colitis [221]. 

Loss of the MMR function is mainly caused by inactivating mutations in the *MLH1*, *MSH2*, *MSH3*, *MSH6*, and *PMS1/2* genes. Additionally, epigenetic changes, e.g., hypermethylation of the *MLH1* promoter, may also be involved in silencing of gene expression of some MMR pathway components (reviewed in Reference [222]). Colorectal tumors associated with MMR deficiency exhibit several characteristic features such as proximal colon localization, mucinous or undifferentiated phenotype, and lymphocytic infiltrations [223]. In mice, homozygous deletion of the MMR genes is mostly compatible with the animal life; however, inactivation of the genes might result in lymphomas and other tumor types including adenomas formed in all segments of the gastrointestinal tract (the corresponding models of the deficient MMR pathway are listed in Table S7, Supplementary Materials). For example, *Mlh1^−/−^* and *Msh2^−/−^* mice developed tumors predominantly in the small intestine and survived no longer than one year [31,224]. *Msh3^−/−^* mice did not exhibit any cancer predispositions; nevertheless, *Msh6^−/−^* mice developed lymphomas of the skin and uterine carcinomas. Interestingly, combination of *Msh6* and *Msh3* null alleles promoted intestinal tumorigenesis [225]. In addition, no neoplastic lesions were observed in *Pms1^−/−^* mice; in contrast, *Pms2^−/−^* animals developed lymphomas and sarcomas and died (without any occurrence of intestinal neoplasia) at the age of 17 months [226]. The absence of the *Mlh3* gene product caused MSI accompanied by impaired DNA damage response and tumor development throughout the lower gastrointestinal tract. In these animals, tumor incidence was further increased by a simultaneous germline deletion of the *Pms2* gene; the resulting phenotype then mirrored the situation observed in *Mlh1^−/−^* mice [227]. Mice harboring *Msh2* cKO alleles and *EIIa-Cre* transgene (the transgene allows constitutive gene recombination of floxed sequences in the zygote [228]) recapitulated the phenotype observed in *Msh2^−/−^* mice, i.e., they displayed MMR deficiency and developed intestinal tumors. In contrast, intestinal inactivation of *Msh2* in *Msh2^cKO/cKO^* *Villin-Cre* mice was compatible with near-standard life expectancy. Strikingly, these mice developed intestinal tumors with truncating somatic *Apc* mutations [229]. Intriguingly, somatic mutations truncating Apc were also detected in tumors developed in *Msh2^−/−^* mice [230]. 

It is evident that MMR deficiency leads to increased predisposition of intestinal cells to mutations that further potentiate tumor growth. For example, *Msh2^−/−^* mice harboring the inducible oncogenic *Kras^V12^* allele developed a higher number of colon adenomas when compared to *Msh2^−/−^* animals producing wt *Kras* [231]. Similarly, germline deletion of *Mlh1* or *Msh2* increased colon tumor incidence in *Apc^+/1638N^* and *Apc^+/Min^* mice, respectively [224,230]. Moreover, mutations in the particular “MMR gene” might also influence the way in which the second (wt) *Apc* allele is inactivated. For example, similarly as in the case of Msh2-deficient mice, *Mlh3^−/−^ Apc^+/1638N^* mice showed increased frequency of frameshift mutations in the wt *Apc* allele; however, these frameshift mutations were, in contrast to mutations induced by MSI, in the non-repetitive sequences. Furthermore, combined homozygous deletion of *Mlh3* and *Pms2* caused increased incidence of base substitutions in *Apc*. Moreover, the position of the genetic changes in the wt *Apc* allele was also dependent on which MMR gene was mutated. For example, *Apc* mutations in *Mlh3^−/−^ Pms2^−/−^* or *Mlh1^−/−^* mice occurred preferentially in the mutation hotspot in codons 854, 929, 1211, and 1464 [227,232,233]. In conclusion, although all of the “MMR genes” belong to one signaling pathway, the phenotype caused by their (combined) mutations varies with respect to the genetic change, tumor type, and tumor incidence.

## 9. Future Perspectives

In this review, we summarized some currently available mouse models of intestinal tumorigenesis. We also attempted to assign the models to the recently introduced CMS system used for classification of human CRCs. Although many mouse strains develop different types of neoplasia as a result of a single mutational event, multiple genetic alterations are necessary to obtain a progressed solid tumor in a “reasonable” time period. Since the initial mutation in the majority of human sporadic colorectal carcinomas occur in the *APC* gene, the effect of mutations in other possibly driver genes is often studied on the Apc-deficient genetic background. Alternatively, to mimic human CAC, the gene of interest can be modified in animals with DSS-induced colitis.

In relation to assignment of individual CRCs to one of the CMS groups, the question arises whether such an assignment, which indicates the gene expression profile of the resected tumor, is retained during tumor progression. Numerous experiments showed that combination of multiple genetic changes and the inflammatory response have a profound influence on the gene expression profile and cell composition of the primary lesion. This fact indicates that CMS group “switching” is common. Consequently, the necessity for sequential (multiple) genetic changes (or epigenetic alterations) limits the usage of the mouse cancer models. Nevertheless, there are recent examples showing that these limitations can be overcome. For example, mouse models using sleeping beauty (*SB*) transposon-based insertional mutagenesis allowed simultaneous inactivation of multiple genes. Moreover, usage of the *SB* system in mice that already carried a driver mutation were employed to either study the importance of the order of certain genetic changes, or to detect low-frequency mutations in the genes that cooperate with the particular driver mutation [234,235]. In addition, intestinal organoid cultures were used to introduce multiple genetic alterations into the genome of intestinal epithelium cells. The indisputable advantage of using organoids is the possibility to work with primary human cells obtained directly from the tumor (or healthy) tissue. Moreover, organoid preparation and genetic manipulations are much faster than generation of a new genetically modified mouse strain. For example, in 2015, two laboratories used the CRISPR/Cas9 system to sequentially introduce four mutations in *APC*, *TP53*, *KRAS*, and *SMAD4* genes into human cells growing as colon organoids [236,237]. We anticipate that organoids, although a very suitable in vitro model, do not contain all cell types present in a tumor growing in a particular organ. Thus, conclusions drawn from the results obtained in organoids do not necessarily correspond to the situation in vivo. Nevertheless, to obtain a more comprehensive and detailed picture, the existing mouse cancer models should be more thoroughly characterized. A high-throughput gene expression and proteomic analysis of mouse tumors induced by different genetic alterations would undoubtedly yield more accurate information on the tumor characteristics developed in a given mouse model.

## Figures and Tables

**Table 1:** Biological characteristics of consensus molecular subtype (CMS) groups of colorectal tumors.

CMS1	CMS2	CMS3	CMS4
MSI Immune	Canonical	Metabolic	Mesenchymal
14%	37%	13%	23%
MSI high	MSI negative	Mixed MSI status	MSI low
CIMP high	CIMP negative	CIMP low	CIMP negative
SCNA low	SCNA high	SCNA moderate	SCNA high
BRAF mutations	TP53 mutations	KRAS mutations	TP53 mutations
	epithelial signature	epithelial signature	mesenchymal signature
	Wnt and Myc target genes upregulation	enhanced metabolism	EMT activation and matrix remodeling
immune infiltration			stromal infiltrationTGFβ signaling activation
worse survival after relaps			worse relaps-free and overall survival

*BRAF*, B-Raf proto-oncogene; CIMP, cytosine-phosphate diester-guanine nucleotide (CpG) island methylator phenotype; EMT, epithelial–mesenchymal transition; *KRAS*, Kirsten rat sarcoma viral oncogene homolog; MSI, microsatellite instability; SCNA, somatic copy number alterations; *TP53*, tumor protein 53 (adopted from Reference [24]).

**Table 2:** Selected mouse models suitable for studying tumors belonging to the particular CMS group. Since CMS4 tumors are mainly characterized by activation of the transforming growth factor β (TGFβ) pathway in stromal cells, we did not include any mouse model to this category. It should be noted that mouse strains allowing downregulation of TGFβ signaling are available. However, tumors developed in these mice fit well into the CMS2 group. N/A, not available.

	Generated Allele or Strain Name	Advantages	Disadvantages	Reference
CMS1	*Braf^V600E^*	crypt hyperproliferation, high incidence of tumors, mucinous phenotype	not all the animals develop tumors	[29]
	*Mlh1^-/-^*	100% tumor development within 4 months	tumors develop in many other tissues, short lifespan	[30]
	*Msh2^loxP/loxP^ Villin-Cre*	90 % of mice developed adenomas and adenocarcinomas, tumor formation is restricted to the intestine	mosaic recombination in the tissue	[31]
CMS2	*Apc^Min^*	multiple intestinal tumors, early tumor development, recapitulates human FAP syndrome	relatively rare tumorigenesis in the colon	[32]
	*Apc^cKO/cKO^ Lgr5-EGFP-IRES-CreERT2*	inducible tumor initiation, all tumors develop during the same (and defined) time period	tamoxifen dose-dependent variability of the phenotype	[33]
	*Catnb^+/lox(ex3)^ Krt1-19-Cre*	early tumor development, large amount of tumors, microadenomas in the colon	short lifespan due to extensive tumorigenesis	[34]
	*Apc^Min^ p53^-/-^*	increased number and invasivity of intestinal tumors	tumors develop in many other tissues, short lifespan	[35]
CMS3	*Apc^2lox14/+^ LSL-Kras^G12D^ Rapbp1-Cre*	combination of Apc and Kras mutations, adenomas in the colon	crossbreeding	[36]
	*Apc^Min^ K-ras^Asp12^ Ah-Cre*	increased number of intestinal tumors with higher effect in the colon	crossbreeding	[37]
CMS4	*N/A*			

## Supplementary Materials

The following supplementary tables are available online at https://www.mdpi.com/2073-4425/10/10/788/s1: Table S1: Chemically induced colitis and intestinal tumors; Table S2: Mouse models of hyperactivated Wnt signaling; Table S3: Mouse models of the deregulated Hippo pathway; Table S4: Mouse models of the impaired p53 pathway; Table S5: Mouse strains modeling aberrant EGF signaling; Table S6: Mouse models of impaired TGFβ signaling; Table S7: Mouse models of DNA mismatch repair deficiency.

## References

[1] Siegel R.L., Miller K.D., Jemal A. (2019). Cancer statistics, 2019. CA Cancer J. Clin..

[2] Kinzler K.W., Vogelstein B. (1996). Lessons from hereditary colorectal cancer. Cell.

[3] Jen J., Powell S.M., Papadopoulos N., Smith K.J., Hamilton S.R., Vogelstein B., Kinzler K.W. (1994). Molecular determinants of dysplasia in colorectal lesions. Cancer Res..

[4] Janssen K.P., Alberici P., Fsihi H., Gaspar C., Breukel C., Franken P., Rosty C., Abal M., El Marjou F., Smits R. (2006). APC and oncogenic KRAS are synergistic in enhancing Wnt signaling in intestinal tumor formation and progression. Gastroenterology.

[5] Smith A.J., Stern H.S., Penner M., Hay K., Mitri A., Bapat B.V., Gallinger S. (1994). Somatic APC and K-ras codon 12 mutations in aberrant crypt foci from human colons. Cancer Res..

[6] Rodrigues N.R., Rowan A., Smith M.E., Kerr I.B., Bodmer W.F., Gannon J.V., Lane D.P. (1990). p53 mutations in colorectal cancer. Proc. Natl. Acad. Sci. USA.

[7] Vogelstein B., Fearon E.R., Hamilton S.R., Kern S.E., Preisinger A.C., Leppert M., Nakamura Y., White R., Smits A.M., Bos J.L. (1988). Genetic alterations during colorectal-tumor development. N. Engl. J. Med..

[8] Weinberg R.A. (1989). Oncogenes, antioncogenes, and the molecular bases of multistep carcinogenesis. Cancer Res..

[9] Mattar M.C., Lough D., Pishvaian M.J., Charabaty A. (2011). Current management of inflammatory bowel disease and colorectal cancer. Gastrointest. Cancer Res..

[10] Van Der Kraak L., Gros P., Beauchemin N. (2015). Colitis-associated colon cancer: Is it in your genes?. World J. Gastroenterol..

[11] Brentnall T.A., Crispin D.A., Rabinovitch P.S., Haggitt R.C., Rubin C.E., Stevens A.C., Burmer G.C. (1994). Mutations in the p53 gene: An early marker of neoplastic progression in ulcerative colitis. Gastroenterology.

[12] Shenoy A.K., Fisher R.C., Butterworth E.A., Pi L., Chang L.J., Appelman H.D., Chang M., Scott E.W., Huang E.H. (2012). Transition from colitis to cancer: High Wnt activity sustains the tumor-initiating potential of colon cancer stem cell precursors. Cancer Res..

[13] Yaeger R., Shah M.A., Miller V.A., Kelsen J.R., Wang K., Heins Z.J., Ross J.S., He Y., Sanford E., Yantiss R.K. (2016). Genomic Alterations Observed in Colitis-Associated Cancers Are Distinct From Those Found in Sporadic Colorectal Cancers and Vary by Type of Inflammatory Bowel Disease. Gastroenterology.

[14] Ullman T.A., Itzkowitz S.H. (2011). Intestinal inflammation and cancer. Gastroenterology.

[15] Andreyev H.J., Norman A.R., Cunningham D., Oates J.R., Clarke P.A. (1998). Kirsten ras mutations in patients with colorectal cancer: The multicenter “RASCAL” study. J. Natl. Cancer Inst..

[16] Smith G., Carey F.A., Beattie J., Wilkie M.J., Lightfoot T.J., Coxhead J., Garner R.C., Steele R.J., Wolf C.R. (2002). Mutations in APC, Kirsten-ras, and p53—Alternative genetic pathways to colorectal cancer. Proc. Natl. Acad. Sci. USA.

[17] Robles A.I., Traverso G., Zhang M., Roberts N.J., Khan M.A., Joseph C., Lauwers G.Y., Selaru F.M., Popoli M., Pittman M.E. (2016). Whole-Exome Sequencing Analyses of Inflammatory Bowel Disease-Associated Colorectal Cancers. Gastroenterology.

[18] Budinska E., Popovici V., Tejpar S., D’Ario G., Lapique N., Sikora K.O., Di Narzo A.F., Yan P., Hodgson J.G., Weinrich S. (2013). Gene expression patterns unveil a new level of molecular heterogeneity in colorectal cancer. J. Pathol..

[19] Marisa L., de Reynies A., Duval A., Selves J., Gaub M.P., Vescovo L., Etienne-Grimaldi M.C., Schiappa R., Guenot D., Ayadi M. (2013). Gene expression classification of colon cancer into molecular subtypes: Characterization, validation, and prognostic value. PLoS Med..

[20] Roepman P., Schlicker A., Tabernero J., Majewski I., Tian S., Moreno V., Snel M.H., Chresta C.M., Rosenberg R., Nitsche U. (2014). Colorectal cancer intrinsic subtypes predict chemotherapy benefit, deficient mismatch repair and epithelial-to-mesenchymal transition. Int. J. Cancer.

[21] De Sousa E.M.F., Wang X., Jansen M., Fessler E., Trinh A., de Rooij L.P., de Jong J.H., de Boer O.J., van Leersum R., Bijlsma M.F. (2013). Poor-prognosis colon cancer is defined by a molecularly distinct subtype and develops from serrated precursor lesions. Nat. Med..

[22] Sadanandam A., Lyssiotis C.A., Homicsko K., Collisson E.A., Gibb W.J., Wullschleger S., Ostos L.C., Lannon W.A., Grotzinger C., Del Rio M. (2013). A colorectal cancer classification system that associates cellular phenotype and responses to therapy. Nat. Med..

[23] Schlicker A., Beran G., Chresta C.M., McWalter G., Pritchard A., Weston S., Runswick S., Davenport S., Heathcote K., Castro D.A. (2012). Subtypes of primary colorectal tumors correlate with response to targeted treatment in colorectal cell lines. BMC Med. Genom..

[24] Guinney J., Dienstmann R., Wang X., de Reynies A., Schlicker A., Soneson C., Marisa L., Roepman P., Nyamundanda G., Angelino P. (2015). The consensus molecular subtypes of colorectal cancer. Nat. Med..

[25] Ning C., Li Y.Y., Wang Y., Han G.C., Wang R.X., Xiao H., Li X.Y., Hou C.M., Ma Y.F., Sheng D.S. (2015). Complement activation promotes colitis-associated carcinogenesis through activating intestinal IL-1β/IL-17A axis. Mucosal Immunol..

[26] Linnekamp J.F., Hooff S.R.V., Prasetyanti P.R., Kandimalla R., Buikhuisen J.Y., Fessler E., Ramesh P., Lee K., Bochove G.G.W., de Jong J.H. (2018). Consensus molecular subtypes of colorectal cancer are recapitulated in in vitro and in vivo models. Cell Death Differ..

[27] Phesse T.J., Durban V.M., Sansom O.J. (2017). Defining key concepts of intestinal and epithelial cancer biology through the use of mouse models. Carcinogenesis.

[28] Taketo M.M., Edelmann W. (2009). Mouse models of colon cancer. Gastroenterology.

[29] Park S.Y., Lee H.S., Choe G., Chung J.H., Kim W.H. (2006). Clinicopathological characteristics, microsatellite instability, and expression of mucin core proteins and p53 in colorectal mucinous adenocarcinomas in relation to location. Virchows Arch..

[30] Oshima H., Nakayama M., Han T.S., Naoi K., Ju X., Maeda Y., Robine S., Tsuchiya K., Sato T., Sato H. (2015). Suppressing TGFβ signaling in regenerating epithelia in an inflammatory microenvironment is sufficient to cause invasive intestinal cancer. Cancer Res..

[31] Reitmair A.H., Redston M., Cai J.C., Chuang T.C., Bjerknes M., Cheng H., Hay K., Gallinger S., Bapat B., Mak T.W. (1996). Spontaneous intestinal carcinomas and skin neoplasms in *Msh2*-deficient mice. Cancer Res..

[32] Su L.K., Kinzler K.W., Vogelstein B., Preisinger A.C., Moser A.R., Luongo C., Gould K.A., Dove W.F. (1992). Multiple intestinal neoplasia caused by a mutation in the murine homolog of the APC gene. Science.

[33] Sasai H., Masaki M., Wakitani K. (2000). Suppression of polypogenesis in a new mouse strain with a truncated Apc^∆474^ by a novel COX-2 inhibitor, JTE-522. Carcinogenesis.

[34] Colnot S., Niwa-Kawakita M., Hamard G., Godard C., Le Plenier S., Houbron C., Romagnolo B., Berrebi D., Giovannini M., Perret C. (2004). Colorectal cancers in a new mouse model of familial adenomatous polyposis: Influence of genetic and environmental modifiers. Lab. Investig. J. Tech. Methods Pathol..

[35] Russo A., Bazan V., Iacopetta B., Kerr D., Soussi T., Gebbia N. (2005). The TP53 colorectal cancer international collaborative study on the prognostic and predictive significance of *p53* mutation: Influence of tumor site, type of mutation, and adjuvant treatment. J. Clin. Oncol..

[36] Mercer K., Giblett S., Green S., Lloyd D., DaRocha Dias S., Plumb M., Marais R., Pritchard C. (2005). Expression of endogenous oncogenic V600EB-raf induces proliferation and developmental defects in mice and transformation of primary fibroblasts. Cancer Res..

[37] Samowitz W.S., Sweeney C., Herrick J., Albertsen H., Levin T.R., Murtaugh M.A., Wolff R.K., Slattery M.L. (2005). Poor survival associated with the *BRAF* V600E mutation in microsatellite-stable colon cancers. Cancer Res..

[38] Ito N., Hasegawa R., Sano M., Tamano S., Esumi H., Takayama S., Sugimura T. (1991). A new colon and mammary carcinogen in cooked food, 2-amino-1-methyl-6-phenylimidazo[4,5-b]pyridine (PhIP). Carcinogenesis.

[39] Ochiai M., Imai H., Sugimura T., Nagao M., Nakagama H. (2002). Induction of intestinal tumors and lymphomas in C57BL/6N mice by a food-borne carcinogen, 2-amino-1-methyl-6-phenylimidazo[4,5-b]pyridine. Jpn. J. Cancer Res..

[40] Nakagama H., Nakanishi M., Ochiai M. (2005). Modeling human colon cancer in rodents using a food-borne carcinogen, PhIP. Cancer Sci..

[41] Yang J., Shikata N., Mizuoka H., Tsubura A. (1996). Colon carcinogenesis in shrews by intrarectal infusion of *N*-methyl-*N*-nitrosourea. Cancer Lett..

[42] Rosenberg D.W., Giardina C., Tanaka T. (2009). Mouse models for the study of colon carcinogenesis. Carcinogenesis.

[43] Deschner E.E., Long F.C. (1977). Colonic neoplasms in mice produced with six injections of 1,2-dimethylhydrazine. Oncology.

[44] Maltzman T., Whittington J., Driggers L., Stephens J., Ahnen D. (1997). AOM-induced mouse colon tumors do not express full-length APC protein. Carcinogenesis.

[45] Takahashi M., Nakatsugi S., Sugimura T., Wakabayashi K. (2000). Frequent mutations of the β-catenin gene in mouse colon tumors induced by azoxymethane. Carcinogenesis.

[46] Vivona A.A., Shpitz B., Medline A., Bruce W.R., Hay K., Ward M.A., Stern H.S., Gallinger S. (1993). K-ras mutations in aberrant crypt foci, adenomas and adenocarcinomas during azoxymethane-induced colon carcinogenesis. Carcinogenesis.

[47] Wang Q.S., Papanikolaou A., Sabourin C.L., Rosenberg D.W. (1998). Altered expression of cyclin D1 and cyclin-dependent kinase 4 in azoxymethane-induced mouse colon tumorigenesis. Carcinogenesis.

[48] Chen J., Huang X.F. (2009). The signal pathways in azoxymethane-induced colon cancer and preventive implications. Cancer Biol. Ther..

[49] Waaler J., Machon O., Tumova L., Dinh H., Korinek V., Wilson S.R., Paulsen J.E., Pedersen N.M., Eide T.J., Machonova O. (2012). A novel tankyrase inhibitor decreases canonical Wnt signaling in colon carcinoma cells and reduces tumor growth in conditional APC mutant mice. Cancer Res..

[50] Bissahoyo A., Pearsall R.S., Hanlon K., Amann V., Hicks D., Godfrey V.L., Threadgill D.W. (2005). Azoxymethane is a genetic background-dependent colorectal tumor initiator and promoter in mice: Effects of dose, route, and diet. Toxicol. Sci..

[51] Greten F.R., Eckmann L., Greten T.F., Park J.M., Li Z.W., Egan L.J., Kagnoff M.F., Karin M. (2004). IKKβ links inflammation and tumorigenesis in a mouse model of colitis-associated cancer. Cell.

[52] Neufert C., Becker C., Neurath M.F. (2007). An inducible mouse model of colon carcinogenesis for the analysis of sporadic and inflammation-driven tumor progression. Nat. Protoc..

[53] Tanaka T., Kohno H., Suzuki R., Yamada Y., Sugie S., Mori H. (2003). A novel inflammation-related mouse colon carcinogenesis model induced by azoxymethane and dextran sodium sulfate. Cancer Sci..

[54] De Robertis M., Massi E., Poeta M.L., Carotti S., Morini S., Cecchetelli L., Signori E., Fazio V.M. (2011). The AOM/DSS murine model for the study of colon carcinogenesis: From pathways to diagnosis and therapy studies. J. Carcinog..

[55] Aoki K., Taketo M.M. (2007). Adenomatous polyposis coli (APC): A multi-functional tumor suppressor gene. J. Cell Sci..

[56] Valenta T., Hausmann G., Basler K. (2012). The many faces and functions of β-catenin. EMBO J..

[57] Kimelman D., Xu W. (2006). β-catenin destruction complex: Insights and questions from a structural perspective. Oncogene.

[58] Stamos J.L., Weis W.I. (2013). The β-catenin destruction complex. Cold Spring Harb. Perspect. Biol..

[59] Saito-Diaz K., Chen T.W., Wang X., Thorne C.A., Wallace H.A., Page-McCaw A., Lee E. (2013). The way Wnt works: Components and mechanism. Growth Factors.

[60] He T.C., Sparks A.B., Rago C., Hermeking H., Zawel L., da Costa L.T., Morin P.J., Vogelstein B., Kinzler K.W. (1998). Identification of c-MYC as a target of the APC pathway. Science.

[61] Shtutman M., Zhurinsky J., Simcha I., Albanese C., D’Amico M., Pestell R., Ben-Ze’ev A. (1999). The cyclin D1 gene is a target of the β-catenin/LEF-1 pathway. Proc. Natl. Acad. Sci. USA.

[62] Wielenga V.J., Smits R., Korinek V., Smit L., Kielman M., Fodde R., Clevers H., Pals S.T. (1999). Expression of CD44 in Apc and Tcf mutant mice implies regulation by the WNT pathway. Am. J. Pathol..

[63] Coppede F., Lopomo A., Spisni R., Migliore L. (2014). Genetic and epigenetic biomarkers for diagnosis, prognosis and treatment of colorectal cancer. World J. Gastroenterol..

[64] Segditsas S., Tomlinson I. (2006). Colorectal cancer and genetic alterations in the Wnt pathway. Oncogene.

[65] Shimizu Y., Ikeda S., Fujimori M., Kodama S., Nakahara M., Okajima M., Asahara T. (2002). Frequent alterations in the Wnt signaling pathway in colorectal cancer with microsatellite instability. Genes Chromosomes Cancer.

[66] Mazzoni S.M., Fearon E.R. (2014). AXIN1 and AXIN2 variants in gastrointestinal cancers. Cancer Lett..

[67] Nishisho I., Nakamura Y., Miyoshi Y., Miki Y., Ando H., Horii A., Koyama K., Utsunomiya J., Baba S., Hedge P. (1991). Mutations of chromosome 5q21 genes in FAP and colorectal cancer patients. Science.

[68] Groden J., Thliveris A., Samowitz W., Carlson M., Gelbert L., Albertsen H., Joslyn G., Stevens J., Spirio L., Robertson M. (1991). Identification and characterization of the familial adenomatous polyposis coli gene. Cell.

[69] Galiatsatos P., Foulkes W.D. (2006). Familial adenomatous polyposis. Am. J. Gastroenterol..

[70] Rubinfeld B., Albert I., Porfiri E., Fiol C., Munemitsu S., Polakis P. (1996). Binding of GSK3β to the APC-β-catenin complex and regulation of complex assembly. Science.

[71] Behrens J., Jerchow B.A., Wurtele M., Grimm J., Asbrand C., Wirtz R., Kuhl M., Wedlich D., Birchmeier W. (1998). Functional interaction of an axin homolog, conductin, with β-catenin, APC, and GSK3β. Science.

[72] Miyoshi Y., Nagase H., Ando H., Horii A., Ichii S., Nakatsuru S., Aoki T., Miki Y., Mori T., Nakamura Y. (1992). Somatic mutations of the APC gene in colorectal tumors: Mutation cluster region in the APC gene. Hum. Mol. Genet..

[73] Miyaki M., Konishi M., Kikuchi-Yanoshita R., Enomoto M., Igari T., Tanaka K., Muraoka M., Takahashi H., Amada Y., Fukayama M. (1994). Characteristics of somatic mutation of the adenomatous polyposis coli gene in colorectal tumors. Cancer Res..

[74] Hayashi S., Rubinfeld B., Souza B., Polakis P., Wieschaus E., Levine A.J. (1997). A *Drosophila* homolog of the tumor suppressor gene adenomatous polyposis coli down-regulates β-catenin but its zygotic expression is not essential for the regulation of Armadillo. Proc. Natl. Acad. Sci. USA.

[75] Moser A.R., Pitot H.C., Dove W.F. (1990). A dominant mutation that predisposes to multiple intestinal neoplasia in the mouse. Science.

[76] Moser A.R., Mattes E.M., Dove W.F., Lindstrom M.J., Haag J.D., Gould M.N. (1993). ApcMin, a mutation in the murine Apc gene, predisposes to mammary carcinomas and focal alveolar hyperplasias. Proc. Natl. Acad. Sci. USA.

[77] Tomita H., Yamada Y., Oyama T., Hata K., Hirose Y., Hara A., Kunisada T., Sugiyama Y., Adachi Y., Linhart H. (2007). Development of gastric tumors in *Apc*^Min/+^ mice by the activation of the β-catenin/Tcf signaling pathway. Cancer Res..

[78] Svendsen C., Alexander J., Knutsen H.K., Husoy T. (2011). The min mouse on FVB background: Susceptibility to spontaneous and carcinogen-induced intestinal tumourigenesis. Anticancer Res..

[79] Sodring M., Gunnes G., Paulsen J.E. (2016). Spontaneous initiation, promotion and progression of colorectal cancer in the novel A/J Min/+ mouse. Int. J. Cancer.

[80] Cooper H.S., Chang W.C., Coudry R., Gary M.A., Everley L., Spittle C.S., Wang H., Litwin S., Clapper M.L. (2005). Generation of a unique strain of multiple intestinal neoplasia (*Apc^+/Min-FCCC^*) mice with significantly increased numbers of colorectal adenomas. Mol. Carcinog..

[81] Bashir O., FitzGerald A.J., Goodlad R.A. (2004). Both suboptimal and elevated vitamin intake increase intestinal neoplasia and alter crypt fission in the *Apc*^Min/+^ mouse. Carcinogenesis.

[82] Lawrance A.K., Deng L., Brody L.C., Finnell R.H., Shane B., Rozen R. (2007). Genetic and nutritional deficiencies in folate metabolism influence tumorigenicity in *Apc*^min/+^ mice. J. Nutr. Biochem..

[83] Mutanen M., Pajari A.M., Oikarinen S.I. (2000). Beef induces and rye bran prevents the formation of intestinal polyps in *Apc*^Min^ mice: Relation to β-catenin and PKC isozymes. Carcinogenesis.

[84] Yang K., Lamprecht S.A., Shinozaki H., Fan K., Yang W., Newmark H.L., Kopelovich L., Edelmann W., Jin B., Gravaghi C. (2008). Dietary calcium and cholecalciferol modulate cyclin D1 expression, apoptosis, and tumorigenesis in intestine of *adenomatous polyposis coli^1638N/+^* mice. J. Nutr..

[85] Kwong L.N., Dove W.F. (2009). APC and its modifiers in colon cancer. Adv. Exp. Med. Biol..

[86] Lamlum H., Ilyas M., Rowan A., Clark S., Johnson V., Bell J., Frayling I., Efstathiou J., Pack K., Payne S. (1999). The type of somatic mutation at APC in familial adenomatous polyposis is determined by the site of the germline mutation: A new facet to Knudson’s ‘two-hit’ hypothesis. Nat. Med..

[87] Sieber O.M., Heinimann K., Gorman P., Lamlum H., Crabtree M., Simpson C.A., Davies D., Neale K., Hodgson S.V., Roylance R.R. (2002). Analysis of chromosomal instability in human colorectal adenomas with two mutational hits at APC. Proc. Natl. Acad. Sci. USA.

[88] Lewis A., Segditsas S., Deheragoda M., Pollard P., Jeffery R., Nye E., Lockstone H., Davis H., Clark S., Stamp G. (2010). Severe polyposis in Apc^1322T^ mice is associated with submaximal Wnt signalling and increased expression of the stem cell marker *Lgr5*. Gut.

[89] Pollard P., Deheragoda M., Segditsas S., Lewis A., Rowan A., Howarth K., Willis L., Nye E., McCart A., Mandir N. (2009). The *Apc^1322T^* mouse develops severe polyposis associated with submaximal nuclear β-catenin expression. Gastroenterology.

[90] Bakker E.R., Hoekstra E., Franken P.F., Helvensteijn W., van Deurzen C.H., van Veelen W., Kuipers E.J., Smits R. (2013). β-Catenin signaling dosage dictates tissue-specific tumor predisposition in Apc-driven cancer. Oncogene.

[91] Quesada C.F., Kimata H., Mori M., Nishimura M., Tsuneyoshi T., Baba S. (1998). Piroxicam and acarbose as chemopreventive agents for spontaneous intestinal adenomas in APC gene 1309 knockout mice. Jpn. J. Cancer Res..

[92] Niho N., Takahashi M., Kitamura T., Shoji Y., Itoh M., Noda T., Sugimura T., Wakabayashi K. (2003). Concomitant suppression of hyperlipidemia and intestinal polyp formation in Apc-deficient mice by peroxisome proliferator-activated receptor ligands. Cancer Res..

[93] Deka J., Kuhlmann J., Muller O. (1998). A domain within the tumor suppressor protein APC shows very similar biochemical properties as the microtubule-associated protein tau. Eur. J. Biochem..

[94] Lewis A., Davis H., Deheragoda M., Pollard P., Nye E., Jeffery R., Segditsas S., East P., Poulsom R., Stamp G. (2012). The C-terminus of Apc does not influence intestinal adenoma development or progression. J. Pathol..

[95] Fodde R., Edelmann W., Yang K., van Leeuwen C., Carlson C., Renault B., Breukel C., Alt E., Lipkin M., Khan P.M. (1994). A targeted chain-termination mutation in the mouse Apc gene results in multiple intestinal tumors. Proc. Natl. Acad. Sci. USA.

[96] Smits R., van der Houven van Oordt W., Luz A., Zurcher C., Jagmohan-Changur S., Breukel C., Khan P.M., Fodde R. (1998). *Apc^1638N^*: A mouse model for familial adenomatous polyposis-associated desmoid tumors and cutaneous cysts. Gastroenterology.

[97] Caspari R., Olschwang S., Friedl W., Mandl M., Boisson C., Boker T., Augustin A., Kadmon M., Moslein G., Thomas G. (1995). Familial adenomatous polyposis: Desmoid tumours and lack of ophthalmic lesions (CHRPE) associated with APC mutations beyond codon 1444. Hum. Mol. Genet..

[98] Davies D.R., Armstrong J.G., Thakker N., Horner K., Guy S.P., Clancy T., Sloan P., Blair V., Dodd C., Warnes T.W. (1995). Severe Gardner syndrome in families with mutations restricted to a specific region of the APC gene. Am. J. Hum. Genet..

[99] Ikenoue T., Yamaguchi K., Komura M., Imoto S., Yamaguchi R., Shimizu E., Kasuya S., Shibuya T., Hatakeyama S., Miyano S. (2015). Attenuated familial adenomatous polyposis with desmoids caused by an APC mutation. Hum. Genome Var..

[100] Wang T., Onouchi T., Yamada N.O., Matsuda S., Senda T. (2017). A disturbance of intestinal epithelial cell population and kinetics in APC1638T mice. Med. Mol. Morphol..

[101] Smits R., Kielman M.F., Breukel C., Zurcher C., Neufeld K., Jagmohan-Changur S., Hofland N., van Dijk J., White R., Edelmann W. (1999). *Apc*1638T: A mouse model delineating critical domains of the adenomatous polyposis coli protein involved in tumorigenesis and development. Genes Dev..

[102] Xu Q., Wang Y.S., Dabdoub A., Smallwood P.M., Williams J., Woods C., Kelley M.W., Jiang L., Tasman W., Zhang K. (2004). Vascular development in the retina and inner ear: Control by Norrin and Frizzled-4, a high-affinity ligand-receptor pair. Cell.

[103] Gaspar C., Franken P., Molenaar L., Breukel C., van der Valk M., Smits R., Fodde R. (2009). A targeted constitutive mutation in the APC tumor suppressor gene underlies mammary but not intestinal tumorigenesis. PLoS Genet..

[104] Crist R.C., Roth J.J., Baran A.A., McEntee B.J., Siracusa L.D., Buchberg A.M. (2010). The armadillo repeat domain of Apc suppresses intestinal tumorigenesis. Mamm. Genome Off. J. Int. Mamm. Genome Soc..

[105] Oshima M., Oshima H., Kitagawa K., Kobayashi M., Itakura C., Taketo M. (1995). Loss of Apc heterozygosity and abnormal tissue building in nascent intestinal polyps in mice carrying a truncated Apc gene. Proc. Natl. Acad. Sci. USA.

[106] Shibata H., Toyama K., Shioya H., Ito M., Hirota M., Hasegawa S., Matsumoto H., Takano H., Akiyama T., Toyoshima K. (1997). Rapid colorectal adenoma formation initiated by conditional targeting of the Apc gene. Science.

[107] Kuraguchi M., Wang X.P., Bronson R.T., Rothenberg R., Ohene-Baah N.Y., Lund J.J., Kucherlapati M., Maas R.L., Kucherlapati R. (2006). Adenomatous polyposis coli (APC) is required for normal development of skin and thymus. PLoS Genet..

[108] Colnot S., Decaens T., Niwa-Kawakita M., Godard C., Hamard G., Kahn A., Giovannini M., Perret C. (2004). Liver-targeted disruption of Apc in mice activates β-catenin signaling and leads to hepatocellular carcinomas. Proc. Natl. Acad. Sci. USA.

[109] El Marjou F., Janssen K.P., Chang B.H., Li M., Hindie V., Chan L., Louvard D., Chambon P., Metzger D., Robine S. (2004). Tissue-specific and inducible Cre-mediated recombination in the gut epithelium. Genesis.

[110] Barker N., van Es J.H., Kuipers J., Kujala P., van den Born M., Cozijnsen M., Haegebarth A., Korving J., Begthel H., Peters P.J. (2007). Identification of stem cells in small intestine and colon by marker gene *Lgr5*. Nature.

[111] Horazna M., Janeckova L., Svec J., Babosova O., Hrckulak D., Vojtechova M., Galuskova K., Sloncova E., Kolar M., Strnad H. (2019). Msx1 loss suppresses formation of the ectopic crypts developed in the Apc-deficient small intestinal epithelium. Sci. Rep..

[112] Robanus-Maandag E.C., Koelink P.J., Breukel C., Salvatori D.C., Jagmohan-Changur S.C., Bosch C.A., Verspaget H.W., Devilee P., Fodde R., Smits R. (2010). A new conditional Apc-mutant mouse model for colorectal cancer. Carcinogenesis.

[113] Cheung A.F., Carter A.M., Kostova K.K., Woodruff J.F., Crowley D., Bronson R.T., Haigis K.M., Jacks T. (2010). Complete deletion of Apc results in severe polyposis in mice. Oncogene.

[114] Gao C., Wang Y.M., Broaddus R., Sun L.H., Xue F.X., Zhang W. (2018). Exon 3 mutations of CTNNB1 drive tumorigenesis: A review. Oncotarget.

[115] Kim S., Jeong S. (2019). Mutation Hotspots in the β-Catenin Gene: Lessons from the Human Cancer Genome Databases. Mol. Cells.

[116] Harada N., Tamai Y., Ishikawa T., Sauer B., Takaku K., Oshima M., Taketo M.M. (1999). Intestinal polyposis in mice with a dominant stable mutation of the β-catenin gene. EMBO J..

[117] Kriz V., Korinek V. (2018). Wnt, RSPO and Hippo Signalling in the Intestine and Intestinal Stem Cells. Genes.

[118] Seshagiri S., Stawiski E.W., Durinck S., Modrusan Z., Storm E.E., Conboy C.B., Chaudhuri S., Guan Y., Janakiraman V., Jaiswal B.S. (2012). Recurrent R-spondin fusions in colon cancer. Nature.

[119] Hashimoto T., Ogawa R., Yoshida H., Taniguchi H., Kojima M., Saito Y., Sekine S. (2019). EIF3E-RSPO2 and PIEZO1-RSPO2 fusions in colorectal traditional serrated adenoma. Histopathology.

[120] Hilkens J., Timmer N.C., Boer M., Ikink G.J., Schewe M., Sacchetti A., Koppens M.A.J., Song J.Y., Bakker E.R.M. (2017). RSPO3 expands intestinal stem cell and niche compartments and drives tumorigenesis. Gut.

[121] Han T., Schatoff E.M., Murphy C., Zafra M.P., Wilkinson J.E., Elemento O., Dow L.E. (2017). R-Spondin chromosome rearrangements drive Wnt-dependent tumour initiation and maintenance in the intestine. Nat. Commun..

[122] Zhao B., Tumaneng K., Guan K.L. (2011). The Hippo pathway in organ size control, tissue regeneration and stem cell self-renewal. Nat. Cell Boil..

[123] Yu F.X., Meng Z., Plouffe S.W., Guan K.L. (2015). Hippo pathway regulation of gastrointestinal tissues. Annu. Rev. Physiol..

[124] Wang L., Shi S., Guo Z., Zhang X., Han S., Yang A., Wen W., Zhu Q. (2013). Overexpression of YAP and TAZ is an independent predictor of prognosis in colorectal cancer and related to the proliferation and metastasis of colon cancer cells. PLoS ONE.

[125] Yuen H.F., McCrudden C.M., Huang Y.H., Tham J.M., Zhang X., Zeng Q., Zhang S.D., Hong W. (2013). TAZ expression as a prognostic indicator in colorectal cancer. PLoS ONE.

[126] Avruch J., Zhou D., Bardeesy N. (2012). YAP oncogene overexpression supercharges colon cancer proliferation. Cell Cycle.

[127] Cho S.Y., Gwak J.W., Shin Y.C., Moon D., Ahn J., Sol H.W., Kim S., Kim G., Shin H.M., Lee K.H. (2018). Expression of Hippo pathway genes and their clinical significance in colon adenocarcinoma. Oncol. Lett..

[128] Wang Q., Gao X., Yu T., Yuan L., Dai J., Wang W., Chen G., Jiao C., Zhou W., Huang Q. (2018). REGγ Controls Hippo Signaling and Reciprocal NF-κB-YAP Regulation to Promote Colon Cancer. Clin. Cancer Res..

[129] Camargo F.D., Gokhale S., Johnnidis J.B., Fu D., Bell G.W., Jaenisch R., Brummelkamp T.R. (2007). YAP1 increases organ size and expands undifferentiated progenitor cells. Curr. Boil. CB.

[130] Barry E.R., Morikawa T., Butler B.L., Shrestha K., de la Rosa R., Yan K.S., Fuchs C.S., Magness S.T., Smits R., Ogino S. (2013). Restriction of intestinal stem cell expansion and the regenerative response by YAP. Nature.

[131] Zhou D., Zhang Y., Wu H., Barry E., Yin Y., Lawrence E., Dawson D., Willis J.E., Markowitz S.D., Camargo F.D. (2011). Mst1 and Mst2 protein kinases restrain intestinal stem cell proliferation and colonic tumorigenesis by inhibition of Yes-associated protein (Yap) overabundance. Proc. Natl. Acad. Sci. USA.

[132] Cai J., Zhang N., Zheng Y., de Wilde R.F., Maitra A., Pan D. (2010). The Hippo signaling pathway restricts the oncogenic potential of an intestinal regeneration program. Genes Dev..

[133] Muller P.A., Vousden K.H. (2014). Mutant p53 in cancer: New functions and therapeutic opportunities. Cancer Cell.

[134] Oliner J.D., Pietenpol J.A., Thiagalingam S., Gyuris J., Kinzler K.W., Vogelstein B. (1993). Oncoprotein MDM2 conceals the activation domain of tumour suppressor p53. Nature.

[135] Amaral J.D., Xavier J.M., Steer C.J., Rodrigues C.M. (2010). The role of p53 in apoptosis. Discov. Med..

[136] Abukhdeir A.M., Park B.H. (2008). P21 and p27: Roles in carcinogenesis and drug resistance. Expert Rev. Mol. Med..

[137] Baker S.J., Preisinger A.C., Jessup J.M., Paraskeva C., Markowitz S., Willson J.K., Hamilton S., Vogelstein B. (1990). p53 gene mutations occur in combination with 17p allelic deletions as late events in colorectal tumorigenesis. Cancer Res..

[138] Hainaut P., Hollstein M. (2000). p53 and human cancer: The first ten thousand mutations. Adv. Cancer Res..

[139] Lopez I., Oliveira L.P., Tucci P., Alvarez-Valin F., Coudry R.A., Marin M. (2012). Different mutation profiles associated to P53 accumulation in colorectal cancer. Gene.

[140] Li X.L., Zhou J., Chen Z.R., Chng W.J. (2015). P53 mutations in colorectal cancer—Molecular pathogenesis and pharmacological reactivation. World J. Gastroenterol..

[141] Ogino S., Nosho K., Shima K., Baba Y., Irahara N., Kirkner G.J., Hazra A., De Vivo I., Giovannucci E.L., Meyerhardt J.A. (2009). p21 expression in colon cancer and modifying effects of patient age and body mass index on prognosis. Cancer Epidemiol. Biomark. Prev..

[142] Ogino S., Kawasaki T., Kirkner G.J., Ogawa A., Dorfman I., Loda M., Fuchs C.S. (2006). Down-regulation of p21 (CDKN1A/CIP1) is inversely associated with microsatellite instability and CpG island methylator phenotype (CIMP) in colorectal cancer. J. Pathol..

[143] Jacks T., Remington L., Williams B.O., Schmitt E.M., Halachmi S., Bronson R.T., Weinberg R.A. (1994). Tumor spectrum analysis in *p53*-mutant mice. Curr. Boil. CB.

[144] Lang G.A., Iwakuma T., Suh Y.A., Liu G., Rao V.A., Parant J.M., Valentin-Vega Y.A., Terzian T., Caldwell L.C., Strong L.C. (2004). Gain of function of a p53 hot spot mutation in a mouse model of Li-Fraumeni syndrome. Cell.

[145] Halberg R.B., Katzung D.S., Hoff P.D., Moser A.R., Cole C.E., Lubet R.A., Donehower L.A., Jacoby R.F., Dove W.F. (2000). Tumorigenesis in the multiple intestinal neoplasia mouse: Redundancy of negative regulators and specificity of modifiers. Proc. Natl. Acad. Sci. USA.

[146] Funabashi H., Uchida K., Kado S., Matsuoka Y., Ohwaki M. (2001). Establishment of a *Tcrb* and *Trp53* genes deficient mouse strain as an animal model for spontaneous colorectal cancer. Exp. Anim..

[147] Cooks T., Pateras I.S., Tarcic O., Solomon H., Schetter A.J., Wilder S., Lozano G., Pikarsky E., Forshew T., Rosenfeld N. (2013). Mutant p53 prolongs NF-κB activation and promotes chronic inflammation and inflammation-associated colorectal cancer. Cancer Cell.

[148] Chang W.C., Coudry R.A., Clapper M.L., Zhang X., Williams K.L., Spittle C.S., Li T., Cooper H.S. (2007). Loss of p53 enhances the induction of colitis-associated neoplasia by dextran sulfate sodium. Carcinogenesis.

[149] Vyas M., Yang X., Zhang X. (2016). Gastric Hamartomatous Polyps-Review and Update. Clin. Med. Insights Gastroenterol..

[150] Karuman P., Gozani O., Odze R.D., Zhou X.C., Zhu H., Shaw R., Brien T.P., Bozzuto C.D., Ooi D., Cantley L.C. (2001). The Peutz-Jegher gene product LKB1 is a mediator of p53-dependent cell death. Mol. Cell.

[151] Tiainen M., Vaahtomeri K., Ylikorkala A., Makela T.P. (2002). Growth arrest by the LKB1 tumor suppressor: Induction of p21(WAF1/CIP1). Hum. Mol. Genet..

[152] Tiainen M., Ylikorkala A., Makela T.P. (1999). Growth suppression by Lkb1 is mediated by a G_1_ cell cycle arrest. Proc. Natl. Acad. Sci. USA.

[153] Miyoshi H., Nakau M., Ishikawa T.O., Seldin M.F., Oshima M., Taketo M.M. (2002). Gastrointestinal hamartomatous polyposis in Lkb1 heterozygous knockout mice. Cancer Res..

[154] Wei C.J., Amos C.I., Stephens L.C., Campos I., Deng J.M., Behringer R.R., Rashid A., Frazier M.L. (2005). Mutation of *Lkb1* and *p53* genes exert a cooperative effect on tumorigenesis. Cancer Res..

[155] Deng C., Zhang P., Harper J.W., Elledge S.J., Leder P. (1995). Mice lacking p21^CIP1/WAF1^ undergo normal development, but are defective in G1 checkpoint control. Cell.

[156] Brugarolas J., Chandrasekaran C., Gordon J.I., Beach D., Jacks T., Hannon G.J. (1995). Radiation-induced cell cycle arrest compromised by p21 deficiency. Nature.

[157] Martin-Caballero J., Flores J.M., Garcia-Palencia P., Serrano M. (2001). Tumor susceptibility of *p21*^Waf1/Cip1^-deficient mice. Cancer Res..

[158] Poole A.J., Heap D., Carroll R.E., Tyner A.L. (2004). Tumor suppressor functions for the Cdk inhibitor p21 in the mouse colon. Oncogene.

[159] Jackson R.J., Engelman R.W., Coppola D., Cantor A.B., Wharton W., Pledger W.J. (2003). p21^Cip1^ nullizygosity increases tumor metastasis in irradiated mice. Cancer Res..

[160] Yang W.C., Mathew J., Velcich A., Edelmann W., Kucherlapati R., Lipkin M., Yang K., Augenlicht L.H. (2001). Targeted inactivation of the *p21^WAF1/cip1^* gene enhances Apc-initiated tumor formation and the tumor-promoting activity of a Western-style high-risk diet by altering cell maturation in the intestinal mucosal. Cancer Res..

[161] Zirbes T.K., Baldus S.E., Moenig S.P., Nolden S., Kunze D., Shafizadeh S.T., Schneider P.M., Thiele J., Hoelscher A.H., Dienes H.P. (2000). Prognostic impact of p21/waf1/cip1 in colorectal cancer. Int. J. Cancer.

[162] Wee P., Wang Z. (2017). Epidermal Growth Factor Receptor Cell Proliferation Signaling Pathways. Cancers.

[163] Tuveson D.A., Shaw A.T., Willis N.A., Silver D.P., Jackson E.L., Chang S., Mercer K.L., Grochow R., Hock H., Crowley D. (2004). Endogenous oncogenic *K-ras^G12D^* stimulates proliferation and widespread neoplastic and developmental defects. Cancer Cell.

[164] Johnson L., Mercer K., Greenbaum D., Bronson R.T., Crowley D., Tuveson D.A., Jacks T. (2001). Somatic activation of the *K-ras* oncogene causes early onset lung cancer in mice. Nature.

[165] Yamashita N., Minamoto T., Ochiai A., Onda M., Esumi H. (1995). Frequent and characteristic K-*ras* activation and absence of p53 protein accumulation in aberrant crypt foci of the colon. Gastroenterology.

[166] Roerink S.F., Sasaki N., Lee-Six H., Young M.D., Alexandrov L.B., Behjati S., Mitchell T.J., Grossmann S., Lightfoot H., Egan D.A. (2018). Intra-tumour diversification in colorectal cancer at the single-cell level. Nature.

[167] Kavuri S.M., Jain N., Galimi F., Cottino F., Leto S.M., Migliardi G., Searleman A.C., Shen W., Monsey J., Trusolino L. (2015). HER2 activating mutations are targets for colorectal cancer treatment. Cancer Discov..

[168] Dougherty U., Cerasi D., Taylor I., Kocherginsky M., Tekin U., Badal S., Aluri L., Sehdev A., Cerda S., Mustafi R. (2009). Epidermal growth factor receptor is required for colonic tumor promotion by dietary fat in the azoxymethane/dextran sulfate sodium model: Roles of transforming growth factor-α and PTGS2. Clin. Cancer Res..

[169] Dube P.E., Yan F., Punit S., Girish N., McElroy S.J., Washington M.K., Polk D.B. (2012). Epidermal growth factor receptor inhibits colitis-associated cancer in mice. J. Clin. Investig..

[170] Roberts R.B., Min L., Washington M.K., Olsen S.J., Settle S.H., Coffey R.J., Threadgill D.W. (2002). Importance of epidermal growth factor receptor signaling in establishment of adenomas and maintenance of carcinomas during intestinal tumorigenesis. Proc. Natl. Acad. Sci. USA.

[171] Nagahara H., Mimori K., Ohta M., Utsunomiya T., Inoue H., Barnard G.F., Ohira M., Hirakawa K., Mori M. (2005). Somatic mutations of epidermal growth factor receptor in colorectal carcinoma. Clin. Cancer Res..

[172] Moroni M., Veronese S., Benvenuti S., Marrapese G., Sartore-Bianchi A., Di Nicolantonio F., Gambacorta M., Siena S., Bardelli A. (2005). Gene copy number for epidermal growth factor receptor (EGFR) and clinical response to antiEGFR treatment in colorectal cancer: A cohort study. Lancet Oncol..

[173] Brink M., de Goeij A.F., Weijenberg M.P., Roemen G.M., Lentjes M.H., Pachen M.M., Smits K.M., de Bruine A.P., Goldbohm R.A., van den Brandt P.A. (2003). *K-ras* oncogene mutations in sporadic colorectal cancer in The Netherlands Cohort Study. Carcinogenesis.

[174] Haigis K.M., Kendall K.R., Wang Y., Cheung A., Haigis M.C., Glickman J.N., Niwa-Kawakita M., Sweet-Cordero A., Sebolt-Leopold J., Shannon K.M. (2008). Differential effects of oncogenic K-Ras and N-Ras on proliferation, differentiation and tumor progression in the colon. Nat. Genet..

[175] Guerra C., Mijimolle N., Dhawahir A., Dubus P., Barradas M., Serrano M., Campuzano V., Barbacid M. (2003). Tumor induction by an endogenous *K-ras* oncogene is highly dependent on cellular context. Cancer Cell.

[176] Ireland H., Kemp R., Houghton C., Howard L., Clarke A.R., Sansom O.J., Winton D.J. (2004). Inducible Cre-mediated control of gene expression in the murine gastrointestinal tract: Effect of loss of β-catenin. Gastroenterology.

[177] Luo F., Brooks D.G., Ye H., Hamoudi R., Poulogiannis G., Patek C.E., Winton D.J., Arends M.J. (2009). Mutated K-*ras^Asp12^* promotes tumourigenesis in *Apc^Min^* mice more in the large than the small intestines, with synergistic effects between K-*ras* and *Wnt* pathways. Int. J. Exp. Pathol..

[178] Hung K.E., Maricevich M.A., Richard L.G., Chen W.Y., Richardson M.P., Kunin A., Bronson R.T., Mahmood U., Kucherlapati R. (2010). Development of a mouse model for sporadic and metastatic colon tumors and its use in assessing drug treatment. Proc. Natl. Acad. Sci. USA.

[179] Poulin E.J., Bera A.K., Lu J., Lin Y.J., Strasser S.D., Paulo J.A., Huang T.Q., Morales C., Yan W., Cook J. (2019). Tissue-Specific Oncogenic Activity of KRAS^A146T^. Cancer Discov..

[180] Davies H., Bignell G.R., Cox C., Stephens P., Edkins S., Clegg S., Teague J., Woffendin H., Garnett M.J., Bottomley W. (2002). Mutations of the *BRAF* gene in human cancer. Nature.

[181] Rajagopalan H., Bardelli A., Lengauer C., Kinzler K.W., Vogelstein B., Velculescu V.E. (2002). Tumorigenesis—*RAF/RAS* oncogenes and mismatch-repair status. Nature.

[182] Dankort D., Filenova E., Collado M., Serrano M., Jones K., McMahon M. (2007). A new mouse model to explore the initiation, progression, and therapy of *BRAF^V600E^*-induced lung tumors. Genes Dev..

[183] Rad R., Cadinanos J., Rad L., Varela I., Strong A., Kriegl L., Constantino-Casas F., Eser S., Hieber M., Seidler B. (2013). A Genetic Progression Model of Braf^V600E^-Induced Intestinal Tumorigenesis Reveals Targets for Therapeutic Intervention. Cancer Cell.

[184] Tao Y., Kang B., Petkovich D.A., Bhandari Y.R., In J., Stein-O’Brien G., Kong X., Xie W., Zachos N., Maegawa S. (2019). Aging-like Spontaneous Epigenetic Silencing Facilitates Wnt Activation, Stemness, and *Braf*^V600^)-Induced Tumorigenesis. Cancer Cell.

[185] Biemer-Huttmann A.E., Walsh M.D., McGuckin M.A., Simms L.A., Young J., Leggett B.A., Jass J.R. (2000). Mucin core protein expression in colorectal cancers with high levels of microsatellite instability indicates a novel pathway of morphogenesis. Clin. Cancer Res..

[186] Walsh M.D., Clendenning M., Williamson E., Pearson S.A., Walters R.J., Nagler B., Packenas D., Win A.K., Hopper J.L., Jenkins M.A. (2013). Expression of MUC2, MUC5AC, MUC5B, and MUC6 mucins in colorectal cancers and their association with the CpG island methylator phenotype. Mod. Pathol..

[187] Winterford C.M., Walsh M.D., Leggett B.A., Jass J.R. (1999). Ultrastructural localization of epithelial mucin core proteins in colorectal tissues. J. Histochem. Cytochem..

[188] Velcich A., Yang W., Heyer J., Fragale A., Nicholas C., Viani S., Kucherlapati R., Lipkin M., Yang K., Augenlicht L. (2002). Colorectal cancer in mice genetically deficient in the mucin Muc2. Science.

[189] Stambolic V., Suzuki A., de la Pompa J.L., Brothers G.M., Mirtsos C., Sasaki T., Ruland J., Penninger J.M., Siderovski D.P., Mak T.W. (1998). Negative regulation of PKB/Akt-dependent cell survival by the tumor suppressor PTEN. Cell.

[190] Velho S., Oliveira C., Ferreira A., Ferreira A.C., Suriano G., Schwartz S., Duval A., Carneiro F., Machado J.C., Hamelin R. (2005). The prevalence of PIK3CA mutations in gastric and colon cancer. Eur. J. Cancer.

[191] Samuels Y., Waldman T. (2010). Oncogenic mutations of PIK3CA in human cancers. Curr. Top. Microbiol. Immunol..

[192] Mitchell C.B., Phillips W.A. (2019). Mouse Models for Exploring the Biological Consequences and Clinical Significance of PIK3CA Mutations. Biomolecules.

[193] Goel A., Arnold C.N., Niedzwiecki D., Carethers J.M., Dowell J.M., Wasserman L., Compton C., Mayer R.J., Bertagnolli M.M., Boland C.R. (2004). Frequent inactivation of PTEN by promoter hypermethylation in microsatellite instability-high sporadic colorectal cancers. Cancer Res..

[194] Berg M., Danielsen S.A., Ahlquist T., Merok M.A., Agesen T.H., Vatn M.H., Mala T., Sjo O.H., Bakka A., Moberg I. (2010). DNA sequence profiles of the colorectal cancer critical gene set KRAS-BRAF-PIK3CA-PTEN-TP53 related to age at disease onset. PLoS ONE.

[195] Carpten J.D., Faber A.L., Horn C., Donoho G.P., Briggs S.L., Robbins C.M., Hostetter G., Boguslawski S., Moses T.Y., Savage S. (2007). A transforming mutation in the pleckstrin homology domain of AKT1 in cancer. Nature.

[196] Bleeker F.E., Felicioni L., Buttitta F., Lamba S., Cardone L., Rodolfo M., Scarpa A., Leenstra S., Frattini M., Barbareschi M. (2008). *AKT1^E17K^* in human solid tumours. Oncogene.

[197] Xu Y., Pasche B. (2007). TGF-β signaling alterations and susceptibility to colorectal cancer. Hum. Mol. Genet..

[198] Meulmeester E., Ten Dijke P. (2011). The dynamic roles of TGF-β in cancer. J. Pathol..

[199] Lampropoulos P., Zizi-Sermpetzoglou A., Rizos S., Kostakis A., Nikiteas N., Papavassiliou A.G. (2012). TGF-β signalling in colon carcinogenesis. Cancer Lett..

[200] Xie W., Rimm D.L., Lin Y., Shih W.J., Reiss M. (2003). Loss of Smad signaling in human colorectal cancer is associated with advanced disease and poor prognosis. Cancer J..

[201] Fleming N.I., Jorissen R.N., Mouradov D., Christie M., Sakthianandeswaren A., Palmieri M., Day F., Li S., Tsui C., Lipton L. (2013). SMAD2, SMAD3 and SMAD4 mutations in colorectal cancer. Cancer Res..

[202] Miyaki M., Iijima T., Konishi M., Sakai K., Ishii A., Yasuno M., Hishima T., Koike M., Shitara N., Iwama T. (1999). Higher frequency of Smad4 gene mutation in human colorectal cancer with distant metastasis. Oncogene.

[203] Takagi Y., Kohmura H., Futamura M., Kida H., Tanemura H., Shimokawa K., Saji S. (1996). Somatic alterations of the DPC4 gene in human colorectal cancers in vivo. Gastroenterology.

[204] Howe J.R., Roth S., Ringold J.C., Summers R.W., Jarvinen H.J., Sistonen P., Tomlinson I.P., Houlston R.S., Bevan S., Mitros F.A. (1998). Mutations in the *SMAD4/DPC4* gene in juvenile polyposis. Science.

[205] Calon A., Lonardo E., Berenguer-Llergo A., Espinet E., Hernando-Momblona X., Iglesias M., Sevillano M., Palomo-Ponce S., Tauriello D.V., Byrom D. (2015). Stromal gene expression defines poor-prognosis subtypes in colorectal cancer. Nat. Genet..

[206] Nakagawa H., Liyanarachchi S., Davuluri R.V., Auer H., Martin E.W., de la Chapelle A., Frankel W.L. (2004). Role of cancer-associated stromal fibroblasts in metastatic colon cancer to the liver and their expression profiles. Oncogene.

[207] Calon A., Espinet E., Palomo-Ponce S., Tauriello D.V., Iglesias M., Cespedes M.V., Sevillano M., Nadal C., Jung P., Zhang X.H. (2012). Dependency of colorectal cancer on a TGF-β-driven program in stromal cells for metastasis initiation. Cancer Cell.

[208] Kulkarni A.B., Huh C.G., Becker D., Geiser A., Lyght M., Flanders K.C., Roberts A.B., Sporn M.B., Ward J.M., Karlsson S. (1993). Transforming Growth Factor-β-1 Null Mutation in Mice Causes Excessive Inflammatory Response and Early Death. Proc. Natl. Acad. Sci. USA.

[209] Shull M.M., Ormsby I., Kier A.B., Pawlowski S., Diebold R.J., Yin M.Y., Allen R., Sidman C., Proetzel G., Calvin D. (1992). Targeted Disruption of the Mouse Transforming Growth Factor-β-1 Gene Results in Multifocal Inflammatory Disease. Nature.

[210] Engle S.J., Hoying J.B., Boivin G.P., Ormsby I., Gartside P.S., Doetschman T. (1999). Transforming growth factor β1 suppresses nonmetastatic colon cancer at an early stage of tumorigenesis. Cancer Res..

[211] Takaku K., Miyoshi H., Matsunaga A., Oshima M., Sasaki N., Taketo M.M. (1999). Gastric and duodenal polyps in *Smad4* (*Dpc4*) knockout mice. Cancer Res..

[212] Zhu Y., Richardson J.A., Parada L.F., Graff J.M. (1998). *Smad3* mutant mice develop metastatic colorectal cancer. Cell.

[213] Kaiser S., Park Y.K., Franklin J.L., Halberg R.B., Yu M., Jessen W.J., Freudenberg J., Chen X., Haigis K., Jegga A.G. (2007). Transcriptional recapitulation and subversion of embryonic colon development by mouse colon tumor models and human colon cancer. Genome Biol..

[214] Zeng Q., Phukan S., Xu Y., Sadim M., Rosman D.S., Pennison M., Liao J., Yang G.Y., Huang C.C., Valle L. (2009). *Tgfbr1* haploinsufficiency is a potent modifier of colorectal cancer development. Cancer Res..

[215] Alberici P., Jagmohan-Changur S., De Pater E., Van Der Valk M., Smits R., Hohenstein P., Fodde R. (2006). *Smad4* haploinsufficiency in mouse models for intestinal cancer. Oncogene.

[216] Sodir N.M., Chen X., Park R., Nickel A.E., Conti P.S., Moats R., Bading J.R., Shibata D., Laird P.W. (2006). Smad3 deficiency promotes tumorigenesis in the distal colon of *Apc^Min/+^* mice. Cancer Res..

[217] Aguilera O., Fraga M.F., Ballestar E., Paz M.F., Herranz M., Espada J., Garcia J.M., Munoz A., Esteller M., Gonzalez-Sancho J.M. (2006). Epigenetic inactivation of the Wnt antagonist *DICKKOPF-1* (*DKK-1*) gene in human colorectal cancer. Oncogene.

[218] Takaku K., Oshima M., Miyoshi H., Matsui M., Seldin M.F., Taketo M.M. (1998). Intestinal tumorigenesis in compound mutant mice of both *Dpc4* (*Smad4*) and *Apc* genes. Cell.

[219] Hamamoto T., Beppu H., Okada H., Kawabata M., Kitamura T., Miyazono K., Kato M. (2002). Compound disruption of *Smad2* accelerates malignant progression of intestinal tumors in *Apc* knockout mice. Cancer Res..

[220] Kunkel T.A. (2009). Evolving views of DNA replication (in)fidelity. Cold Spring Harb. Symp. Quant. Biol..

[221] O’Sullivan J.N., Bronner M.P., Brentnall T.A., Finley J.C., Shen W.T., Emerson S., Emond M.J., Gollahon K.A., Moskovitz A.H., Crispin D.A. (2002). Chromosomal instability in ulcerative colitis is related to telomere shortening. Nat. Genet..

[222] Poulogiannis G., Frayling I.M., Arends M.J. (2010). DNA mismatch repair deficiency in sporadic colorectal cancer and Lynch syndrome. Histopathology.

[223] Boland C.R., Goel A. (2010). Microsatellite instability in colorectal cancer. Gastroenterology.

[224] Edelmann W., Yang K., Kuraguchi M., Heyer J., Lia M., Kneitz B., Fan K., Brown A.M., Lipkin M., Kucherlapati R. (1999). Tumorigenesis in *Mlh1* and *Mlh1/Apc1638N* mutant mice. Cancer Res..

[225] de Wind N., Dekker M., Claij N., Jansen L., van Klink Y., Radman M., Riggins G., van der Valk M., van’t Wout K., te Riele H. (1999). HNPCC-like cancer predisposition in mice through simultaneous loss of Msh3 and Msh6 mismatch-repair protein functions. Nat. Genet..

[226] Prolla T.A., Baker S.M., Harris A.C., Tsao J.L., Yao X., Bronner C.E., Zheng B., Gordon M., Reneker J., Arnheim N. (1998). Tumour susceptibility and spontaneous mutation in mice deficient in Mlh1, Pms1 and Pms2 DNA mismatch repair. Nat. Genet..

[227] Chen P.C., Dudley S., Hagen W., Dizon D., Paxton L., Reichow D., Yoon S.R., Yang K., Arnheim N., Liskay R.M. (2005). Contributions by MutL homologues *Mlh3* and *Pms2* to DNA mismatch repair and tumor suppression in the mouse. Cancer Res..

[228] Lakso M., Pichel J.G., Gorman J.R., Sauer B., Okamoto Y., Lee E., Alt F.W., Westphal H. (1996). Efficient in vivo manipulation of mouse genomic sequences at the zygote stage. Proc. Natl. Acad. Sci. USA.

[229] Kucherlapati M.H., Lee K., Nguyen A.A., Clark A.B., Hou H., Rosulek A., Li H., Yang K., Fan K., Lipkin M. (2010). An *Msh2* conditional knockout mouse for studying intestinal cancer and testing anticancer agents. Gastroenterology.

[230] Reitmair A.H., Cai J.C., Bjerknes M., Redston M., Cheng H., Pind M.T., Hay K., Mitri A., Bapat B.V., Mak T.W. (1996). MSH2 deficiency contributes to accelerated APC-mediated intestinal tumorigenesis. Cancer Res..

[231] Luo F., Brooks D.G., Ye H., Hamoudi R., Poulogiannis G., Patek C.E., Winton D.J., Arends M.J. (2007). Conditional expression of mutated K-*ras* accelerates intestinal tumorigenesis in *Msh2*-deficient mice. Oncogene.

[232] Kuraguchi M., Edelmann W., Yang K., Lipkin M., Kucherlapati R., Brown A.M. (2000). Tumor-associated *Apc* mutations in *Mlh1^−/−^Apc^1638N^* mice reveal a mutational signature of Mlh1 deficiency. Oncogene.

[233] Kuraguchi M., Yang K., Wong E., Avdievich E., Fan K., Kolodner R.D., Lipkin M., Brown A.M., Kucherlapati R., Edelmann W. (2001). The distinct spectra of tumor-associated *Apc* mutations in mismatch repair-deficient *Apc^1638N^* mice define the roles of MSH3 and MSH6 in DNA repair and intestinal tumorigenesis. Cancer Res..

[234] Takeda H., Rust A.G., Ward J.M., Yew C.C., Jenkins N.A., Copeland N.G. (2016). Sleeping Beauty transposon mutagenesis identifies genes that cooperate with mutant *Smad4* in gastric cancer development. Proc. Natl. Acad. Sci. USA.

[235] Starr T.K., Allaei R., Silverstein K.A., Staggs R.A., Sarver A.L., Bergemann T.L., Gupta M., O’Sullivan M.G., Matise I., Dupuy A.J. (2009). A transposon-based genetic screen in mice identifies genes altered in colorectal cancer. Science.

[236] Drost J., van Jaarsveld R.H., Ponsioen B., Zimberlin C., van Boxtel R., Buijs A., Sachs N., Overmeer R.M., Offerhaus G.J., Begthel H. (2015). Sequential cancer mutations in cultured human intestinal stem cells. Nature.

[237] Matano M., Date S., Shimokawa M., Takano A., Fujii M., Ohta Y., Watanabe T., Kanai T., Sato T. (2015). Modeling colorectal cancer using CRISPR-Cas9-mediated engineering of human intestinal organoids. Nat. Med..

