# Supplementary material for: Human Colorectal Cancer from the Perspective of Mouse Models
Source: Genes (Basel). 2019 Oct 11;10(10):788. doi: 10.3390/genes10100788 (PMC6826908; doi:10.3390/genes10100788)
Supplement: Supplementary file 1 [file genes-10-00788-s001.pdf]

**Table S1: Chemically induced tumorigenesis in mice**

| *Chemical compound* | *Abbreviation* | *Dosage* | *Administration* | *Time* | *Tumor localization in the gut* | *Localization of extraintestinal tumors (if present)* | *Bakground strain* | *Reference* |
|---|---|---|---|---|---|---|---|---|
| 2-amino-1-methyl-6-phenylimidazol[4,5-b]pyridine | PhIP | 300 p.p.m in high fat diet | oral, continuous feeding | 40 weeks | small intestine | | C57BL/6N | [30] |
| N-methyl-N-nitroso-urea | MNU | 1.5 mg/mouse; 16 doses | intrarectal | 31 weeks | colon | uterine squamous cell carcinomas; mesenteric lymph node metastasis | house musk shrew (Jic: SUN) | [32] |
| 1,2-dimethylhydrazine | DMH | 20 mg/kg | subcutaneous | 20-45 weeks | colon | | CF-1 | [34] |
| azoxymethane | AOM | 10 mg/kg; 1-6 doses | intraperitoneal | 12-24 weeks | colon | | AJ, SWR/J, Balb/CJ, CD-1, CBA/J, FVB/N, C57BL/6 | [43] |
| azoxymethane/dextran sulfate sodium salt | AOM/DSS | 10 mg/kg of AOM and 5 days of 1-3 % DSS | intraperitoneal/oral | 10 weeks | colon | | AJ, SWR/J, Balb/CJ, CD-1, CBA/J, FVB/N, C57BL/6 | [43] |

**Table S2: Mouse models of hyperactivated Wnt signaling**

| *Gene name* | *Generated allele* | *Mutation type* | *Outcome of mutation* | *Tumor localization in the gut* | *Tumor count** | *Localization of extraintestinal tumors (if present)* | *Bakground strain* | *Reference* |
|---|---|---|---|---|---|---|---|---|
| *Apc* | $Apc^{\Delta ex1\text{-}15}$ | floxed exon 1-15; *Meox-Cre* | null allele; embryonic recombination | small intestine, occasionally colon | xxx | | C57BL/6 | [107] |
| *Apc* | $Apc^{\Delta 242}$ | β-geo cassette inserted between exon 7 and 8 | truncated Apc protein 241 aa | small intestine | xxx | | C57BL/6J | [96] |
| *Apc* | $Apc^{\Delta 474}$ | NeoR cassette inserted in exon 9; duplication of exons 7-10 | truncated Apc protein 474 aa | small intestine | xxx | mammary adenoacanthoma | C57BL/6 | [97] |
| *Apc* | $Apc^{580D}$ | *Ad-Cre* injected into colorectum | truncated Apc protein 580 aa | colorectum | x | | ND | [99] |
| *Apc* | $Apc^{\Delta 580}$ | *Krt14-Cre* | truncated Apc protein 580 aa in the epithelial cells | small intestine | xxx | squamosus metaplasia in various epithelial tissue | C57BL/6J | [100] |
| *Apc* | $Apc^{\Delta 14}$ | *MeuCre40* | truncated Apc protein 580 aa; embryonic recombination | small intestine, colon | xx; tubular adenomas and invasive carcinomas | | C57BL/6 | [102] |
| *Apc* | $Apc^{\Delta 15}$ | *Tg·Fabpl-Cre* | truncated Apc protein 650 aa in the distal small intestine and colon | small intestine, occasionally colon | xxx | cutaneous follicular cysts, desmoid tumors | C57BL/6 | [106] |
| *Apc* | $Apc^{\Delta 716}$ | NeoR cassette inserted in exon 15 | truncated Apc protein 716 aa | small intestine | xxxx | | C57BL/6J | [98] |
| *Apc* | $Apc^{Min}$ | chemically-induced germinal mutation | truncated Apc protein 850 aa | small intestine, occasionally colon | xxx | stomach, mammary gland | C57BL/6J | [66] |
| *Apc* | $Apc^{Min\text{-}FCCC}$ | chemically-induced germinal mutation | truncated Apc protein 850 aa | small intestine, colon | xxx; adenocarcinomas in colon | stomach, mammary gland | C57BL/6J | [72] |
| *Apc* | $Apc^{1309}$ | | truncated Apc protein 1309 aa | small intestine, occasionally colon | xx | stomach | C57BL/6J | [83] |
| *Apc* | $Apc^{1322T}$ | NeoR cassette inserted in exon 15 | truncated Apc protein 1322 aa | small intestine, occasionally colon | xxxx | | C57BL/6J | [81] |
| *Apc* | $Apc^{1572T}$ | NeoR cassette inserted in exon 15 | truncated Apc protein 1572 aa | | tumor free | mammary adenocarcinomas | C57BL/6J | [95] |
| *Apc* | $Apc^{1638N}$ | NeoR cassette inserted in exon 15 (antisense insertion) | null allele | small instestine | x | cutaneous follicular cysts, desmoid tumors | not clear C57BL/6 or mix | [87] |
| *Apc* | $Apc^{1638T}$ | HygR cassette inserted in exon 15 (sense insertion) | truncated Apc protein 1638 aa | | tumor free | | used C57BL/6J and mixed 129/Ola and C57BL/6 | [88] |
| *Apc* | $Apc^{\Delta SAMP}$ | floxed codons 1322-2006; *Pgk-Cre* | central portion of the Apc gene deleted, while the C-terminus remained intact | small intestine | xxx | | C57BL/6J | [86] |
| *Ctnnb1* | $Catnb^{+/lox(ex3)}$ | floxed exon 3; *Krt1-19-Cre* | stabilized β-catenin in the epithelial cells | small intestine, colon | xxxx; microadenomas in colon | | C57BL/6N | [110] |
| *Ctnnb1* | $Catnb^{+/lox(ex3)}$ | floxed exon 3; *Tg·Fabpl-Cre* | stabilized β-catenin in the distal small intestine and colon | small intestine, colon | xxxx | | C57BL/6N | [110] |
| *Rspo3* | $Rspo3^{inv}$ | *Lgr5-EGFP-IRES-CreERT2* + tamoxifen | conditional *Rspo3* expression from a synthetic CAGGS promoter | small intestine, caecum, colon | x | | FVB | [114] |
| *Rspo3* | *Ptprk(ex1)-Rspo3* | inducible CRISPR/Cas9 + doxocycline | gene fusion Ptprk-Rspo3 | small intestine | displasia and hyperplasia | | mixed 129 and C57BL/6 | [115] |
| *Rspo2* | *Eif3e(ex1)-Rspo2* | inducible CRISPR/Cas9 + doxocycline | gene fusion Eif3e-Rspo2 | small intestine | hyperplasia with limited growth | | mixed 129 and C57BL/6 | [115] |

*) x < 10; xx 10-50; xxx 50-200; xxxx > 200

**Table S3: Mouse models of the deregulated Hippo pathway**

| *Gene name* | *Generated allele* | *Mutation type* | *Outcome of mutation* | *Additional treatment* | *Phenotype in the gut* | *Phenotype in other tissues* | *Bakground strain* | *Reference* |
|---|---|---|---|---|---|---|---|---|
| *Yap1* | *Yap1 $^{S127A}$* | doxycycline-dependent allele | conditionally stabilized Yap protein | | dysplasia and hyperplasia in the small intestine; (colon was not alanyzed) | enlargement of multiple organs | ND | [123] |
| *Yap1* | *Yap1 $^{S127A}$* | *Villin-rtTA* + doxycycline | conditionally stabilized Yap protein in the intestinal epithelium | | degeneration of the small intestinal epithelium; (colon was not alanyzed) | | ND | [124] |
| *Yap1* | *Yap $^{flox/flox}$* | *Villin-Cre* | loss of Yap protein in the intestinal epithelium | chemically induced colitis (DSS) | increased colitis-mediated damage of the colon epithelium | | ND | [126] |
| *Sav1* | *Sav1 $^{flox/flox}$* | *Villin-Cre* | loss of Sav1 in the intestinal epithelium | | hyperplasia in the small intestine and colon | | ND | [126] |
| *Mst1; Mst2* | *Mst1 $^{null}$ /Mst2 $^{f/f}$* | *Villin-Cre* | complete Mst1 loss and Mst2 loss in the intestinal epithelium | | dysplasia in the small intestine and colon; reduced caecum and adenomas in the distal part of colon | | mixed 129/Sv and C57BL/6 | [125] |
| *Regγ* | *Regγ $^{-/-}$* | deletion of exons 5-9 | loss of Regγ protein | chemically induced colitis-associated carcinogenesis (AOM/DSS) | smaller and less tumors than control animals | | C57BL/6 | [122] |

**Table S4: Mouse models of the impaired p53 pathway**

| *Gene name* | *Generated allele* | *Mutation type* | *Outcome of mutation* | *Additional treatment* | *Phenotype in the gut* | *Phenotype in other tissues* | *Bakground strain* | *Reference* |
|---|---|---|---|---|---|---|---|---|
| *Trp53* | *p53* $^{-/-}$ | exons 2-6 replaced by NeoR cassette | loss of p53 protein | | rare intestinal tumors, mice viable up to 6 months | lymphomas, osteosarcomas, adenocarcinomas etc. | mixed 129SV and C57BL/6 | [138] |
| *Trp53* | *p53* $^{+/515A}$ | loxP-PGK-NeoR cassette inserted in intron 4; *CMV-cre* | p53 truncation 515 aa | | | lymphomas, osteosarcomas with high metastases rate | C57BL/6 | [139] |
| *Trp53* | *p53* $^{+/-}$ and *p53* $^{+/515A}$ | | heterozygous loss or truncation of p53 | chemically induced colitis (DSS); chemically induced colitis-associated carcinogenesis (AOM/DSS) | more severe colitis and inflammation-induced carcinogenesis in colon of p53$^{+/515A}$ mice | | C57BL/6 | [142] |
| *Trp53* | *p53* $^{-/-}$ | NeoR cassette inserted in exon 5 | loss of p53 protein | | increased number and invasiveness of intestinal adenomas in *Apc* $^{+/Min}$ mice | | C57BL/6 | [140] |
| *Trp53; Tcr β* | *p53* $^{-/-}$ *Tcrβ* $^{-/-}$ | | loss of p53 in IBD-predisposed mouse | | adenocarcinomas in caecum and dysplasia in colon | tumors of thymus | C57BL/6JCL | [141] |
| *Lkb1* | *Lkb1* $^{-/+}$ | exons 2-4 replaced by NeoR cassette | heterozygous loss of Lkb1 protein | | hamartomatous polyps in stomach (100%) and small intestine (31%), mice viable up to 70 weeks | vascular abnormalities | ND | [148] |
| *Lkb1; Trp53* | *Lkb1* $^{-/+}$ *p53* $^{+/-}$ | Lkb1: floxed exons 2-8; *CMV-cre* Trp53: exons 2-6 replaced by NeoR | heterozygous loss of Lkb1 and p53 | | hamartomatous polyps in stomach and small intestine, mice viable for 10 months | organomegaly in Lkb1$^{+/-}$mice; osteosarcomas and other tumors | 129 | [149] |
| *Cdkn1a* | *p21* $^{-/-}$ | exons 2-3 replaced by PGK-NeoR cassette | loss of p21 protein | chemically induced carcinogenesis (AOM) | enhanced formation of aberrant crypt foci (ACF) through proximal to distal colon | | ND | [153] |
| *Cdkn1a* | *p21* $^{-/-}$ | exons 2 replaced by PGK-NeoR cassette | loss of p21 protein | | altered cell maturation in the intestinal mucosa; increased tumor formation in *Apc* $^{+/1638}$ animals | | ND | [155] |

**Table S5: Mouse strains modeling aberrant EGF signaling**

| *Gene name* | *Generated allele* | *Mutation type* | *Outcome of mutation* | *Additional treatment* | *Phenotype in the gut* | *Phenotype in other tissues (if present)* | *Bakground strain* | *Reference* |
|---|---|---|---|---|---|---|---|---|
| Egfr | $Egfr^{wa2/wa2}$ | missense mutation in tyrosine kinase domain of Egfr | dramatic loss of Egfr kinase activity | chemically induced colitis-associated carcinogenesis (AOM/DSS) | lower incidence of chemically-induced tumors in colon | | mixed C57BL6/J and A/J | [163] |
| Egfr | $Egfr^{wa5/wa5}$ | missense mutation in tyrosine kinase domain of Egfr | kinase-dead Egfr | chemically induced colitis-associated carcinogenesis (AOM/DSS) | enhanced hyperplasia and dysplasia in colon | | C57BL6 | [164] |
| Egfr, Il10 | $Egfr^{wa5/wa5}$ $Il10^{-/-}$ | missense mutation in tyrosine kinase domain of Egfr | kinase-dead Egfr in IBD-predisposed mouse | | increased incidence and progression of tumors in colon | | C57BL6 | [164] |
| Kras | $Kras^{LA1}$ ($Kras^{LA2}$) | insertion of mutant (G12D) exon 1 into Kras gene | stochastic activation of oncogenic Kras (G12D) | | aberrant crypt foci (AFC) in colon without further progression to adenomas; duodenal adenocarcinomas in $Apc^{+/Min}$ and $Trp53^{-/-}$ mice | lung tumors | pure 129 and mixed C57BL6/129 | [159] |
| Kras | $Kras^{G12V}$ *IRESβgeo* | *CMV-Cre; RERTn-CreERT2* + tamoxifen | activation of oncogenic Kras (G12V) | | none | lung tumors | mixed C57BL6/J and 129 | [170] |
| Kras | $LSL\text{-}K\text{-}ras^{G12D}$ | *Fabp-Cre* | activation of oncogenic Kras (G12D) | | hyperproliferation of colonic crypts | | mixed C57BL6 and 129SV | [158] |
| Kras | $LSL\text{-}Kras^{G12D}$ | *Fabp-Cre* | activation of oncogenic Kras (G12D) | | hyperproliferation of colonic crypts; progression of colon adenomas in $Apc^{2lox14/+}$ mice | | ND | [169] |
| Nras | $LSL\text{-}Nras^{G12D}$ | *Fabp-Cre* | activation of oncogenic Nras (G12D) | chemically induced colitis (DSS) | reduced colitis-associated apoptosis | | C57BL6 | [169] |
| Kras | $K\text{-}ras^{Asp12}$ | *Ah-Cre* | activation of oncogenic Kras (Asp12) | β-naphthoflavone treatment | adenomas in the small intestine and colon within two years; increased number of adenomas in $Apc^{+/Min}$ in the small intestine and with higher effect in colon | | FVB/N | [172] |
| Kras | $LSL\text{-}K\text{-}ras^{G12D}$ | *Ad-Cre* injected into colorectum | activation of oncogenic Kras (G12D) | | progression and metastases of $Apc^{cKO/cKO}$ colon adenocarcinomas | | ND | [173] |
| Kras | $Kras^{A146T}$ | *Rapbp1-Cre* | activation of oncogenic Kras (A146T) | | hyperproliferation of colonic crypts milder than in $K\text{-}ras^{G12D}$; progression of colon adenomas in $Apc^{+/2lox14}$ mice | | C57BL6 | [174] |
| Braf | $Braf^{V600E}$ | *Villin-Cre* | activation of oncogenic Braf (V600E) | | hyperproliferation of colonic crypts, higher incidence of tumors | | ND | [180] |
| Muc2 | $Muc2^{-/-}$ | exons 2-4 replaced by PGK-Neo cassette | homozygous loss of Muc2 protein | | adenocarcinomas in small intestine and colon, more than 1.5 tumor per mice after 12 months | | mixed C57BL6J and 129OLA | [186] |

**Table S6: Mouse models of impaired TGFβ signaling**

| *Gene name* | *Generated allele* | *Mutation type* | *Outcome of mutation* | *Additional treatment* | *Tumor localization in the gut* | *Tumor incidence* | *Localization of extraintestinal tumors (if present)* | *Bakground strain* | *Reference* |
|---|---|---|---|---|---|---|---|---|---|
| Tgfb1 | *Tgfb1* $^{-/-}$ *Rag2* $^{-/-}$ | exon 6 replaced by NeoR cassette | loss of Tgfb1 in immunodeficient mice | | caecum, colon | colon carcinomas within 5 months | | mixed 129S6 and CF-1 | [208] |
| Tgfbr2 | *Tgfbr2* $^{E2flox/E2flox}$ | floxed exon 2; *Villin-CreERT2* + tamoxifen | conditional loss of Tgfbr1 in the intestinal epithelium | chemically induced colitis (DSS) | colon | invasive colon carcinomas in 3-8 months | | C57BL/6 | [218] |
| Smad3 | *Smad3* $^{-/-}$ | exon 2 replaced by IRES-LacZ-Neo cassette | loss of Smad3 | | colorectum | invasive tumors in 4-6 months | metastases in lymph nodes | 129/SV | [210] |
| Smad4 | *Smad4* $^{+/-}$ | exon 1 replaced by NeoR cassette | partial loss of Smad4 | | stomach, small intestine | hamartomatous polyps in 1-2 years; more invasive tumors with extensive stromal cell proliferation in *Apc* $^{+/\Delta7106}$ mice | | C57BL/6N | [209, 216] |

**Table S7: Mouse models of DNA mismatch repair deficiency**

| *Gene name* | *Generated allele* | *Mutation type* | *Outcome of mutation* | *Tumor localization in the gut* | *Tumor incidence and life span* | *Localization of extraintestinal tumors (if present)* | *Bakground strain* | *Reference* |
|---|---|---|---|---|---|---|---|---|
| *Mlh1* | *Mlh1* $^{-/-}$ | exon 2 replaced by NeoR cassette | loss of Mlh1 | stomach, small intestine, colon | all mice developed adenomas and adenocarcinomas, viability 4-12 months; increased tumor incidence in *Apc* $^{+/1638N}$ mice | lymphomas, skin tumors and sarcomas | C57BL/6J | [223] |
| *Mlh3* | *Mlh3* $^{-/-}$ | exon 4 replaced by PGK HPRT cassette | loss of Mlh3 | stomach, small intestine, colon, rectum | 50% of mice developed adenomas and adenocarcinomas, viability 15-21 months | lymphomas, basal cell carcinoma of the skin, mammary gland carcinomas, osteosarcomas, testicular cancer, hepatic adenomas | 129 SV/EV | [227] |
| *Mlh3; Pms2* | *Mlh3* $^{-/-}$ *Pms2* $^{-/-}$ | Mlh3: exon 4 replaced by PGK HPRT cassette Pms2: exon 2 of Pms2 replaced by NeoR cassette | simultaneous loss of Mlh3 and Pms2 | stomach, small intestine, colon, rectum | 84% of mice developed adenomas and adenocarcinomas, viability 6-12 months | lymphomas, basal cell carcinoma of the skin, mammary gland carcinomas, osteosarcomas, testicular cancer, hepatic adenomas | 129 SV/EV | [227] |
| *Msh2* | *Msh2* $^{-/-}$ | NeoR cassette inserted in exon 11 | loss of Msh2 | small intestine and colon | 80% of mice developed adenomas and adenocarcinomas, viability 6-11 months; increased tumor incidence in *Apc* $^{+/Min}$ mice | lymphomas | mixed C57BL/6J and 129/OLA | [224, 230] |
| *Msh2* | *Msh2* $^{loxP/loxP}$ | floxed exon 12; *Villin-Cre* | conditional loss of Msh2 in the intestinal epithelium | small intestine and colon | 90% of mice developed adenomas and adenocarcinomas, viability 8-17 months | | mixed C57BL/6J, 129/SV and SJL | [229] |
| *Msh6* | *Msh6* $^{-/-}$ | PuroR cassette inserted in exon 4 | loss of Msh6 | not indicated | rare intestinal tumors (< 10% of mice), viability 6-12 months | lymphomas, epithelial cancers of the uterus and skin | mixed 129/OLA and FVB | [225] |
| *Msh3/6* | *Msh3* $^{-/-}$ *Msh6* $^{-/-}$ | Msh3: HygR cassette inserted in exon 2<br>Msh6: PuroR cassette inserted in exon 4 | simultaneous loss of Msh3 and Msh6 | not indicated | 60% of mice developed intestinal tumors, viability 6-10 months | lymphomas, skin tumors | mixed 129/OLA and FVB | [225] |
